# Imaging features based on CT and MRI for predicting prognosis of patients with intrahepatic cholangiocarcinoma: a single-center study and meta-analysis

**DOI:** 10.1186/s40644-023-00576-5

**Published:** 2023-06-07

**Authors:** Dongwei Sun, Zhenggang XU, Shuya Cao, Huaiyu Wu, Ming LU, Qing Xu, Ke Wang, Guwei Ji

**Affiliations:** 1 Hepatobiliary Center, The First Affiliated Hospital of Nanjing Medical University, 300 Guangzhou Road, Nanjing, 210029 People’s Republic of China; 2 Key Laboratory of Liver Transplantation, Chinese Academy of Medical Sciences, 300 Guangzhou Road, Nanjing, 210029 People’s Republic of China; 3 NHC Key Laboratory of Living Donor Liver Transplantation, Nanjing Medical University, 300 Guangzhou RoadJiangsu Province, Nanjing, People’s Republic of China; 4 Department of Radiology, The First Affiliated Hospital of Nanjing Medical University, Nanjing Jiangsu Province, China

**Keywords:** Intrahepatic cholangiocarcinoma, Imaging feature, Prognosis, Meta-analysis

## Abstract

**Background:**

To evaluate the prognostic role of imaging features based on CT and MRI in intrahepatic cholangiocarcinoma (ICC).

**Methods:**

Two hundred and four patients from a single-center database who underwent radical ICC surgery from 2010 to 2019 were enrolled in the study. Cox proportional hazard model was used for survival analysis of imaging features. A meta-analysis was performed to determine imaging features that predict overall survival (OS) and event-free survival (EFS) in ICC.

**Results:**

In the CT group of the retrospective cohort, tumor multiplicity, infiltrative tumor margin, lymph node metastasis, enhancement pattern in hepatic arterial phase and tumor necrosis correlated with poorer EFS and OS; moreover, enhancing capsules, high carcinoembryonic antigen levels contributed to poor OS. In the MRI group, tumor multiplicity and enhancement pattern were prognostic factors for OS; tumor multiplicity and enhancement pattern resulted in poor EFS. A total of 13 articles containing 1822 patients with ICC were enrolled in the adjusted hazard ratios meta-analysis. The results showed that enhancement pattern and infiltrative tumor margin were predictors of OS and EFS, whereas bile duct invasion was a predictor of OS.

**Conclusions:**

Arterial enhancement patterns and tumor margin status were associated with both OS and EFS of ICC patients following resection.

**Supplementary Information:**

The online version contains supplementary material available at 10.1186/s40644-023-00576-5.

## Introduction

Intrahepatic cholangiocarcinoma (ICC) is the second most common primary liver malignancy, accounting for approximately 10%-15% of all primary liver cancers [1, 2]. The incidence and mortality of ICC continue to increase worldwide [2, 3]. ICC which is defined as a tumor located in the proximal part of the secondary bile duct is further classified into mass-forming, periductal infiltrative, and intraductal growth types based on the morphology and growth pattern of the tumor. Intrahepatic mass-forming cholangiocarcinoma (IMCC) is the most common subtype, constituting approximately 80% of cases, and has the worst prognosis [2, 4–7]. Hepatectomy is deemed to be the possible curative treatment for ICC. Even if the tumor is completely resected, the postoperative outcome of ICC patients remains poor, with a 5-year survival rate of 20%-35%. Moreover, the postoperative recurrence rate is as high as 70% [8, 9]. Recently, advances in neoadjuvant/adjuvant therapy have shown promising results in the preoperative downstaging of ICC as well as survival benefits after radical resection [10–13]. Identifying the prognostic factors of ICC after hepatectomy, determining the ICC patients with a poor postoperative prognosis, and targeting individualized treatment plans are advantageous for patients. Most current ICC staging systems are based on postoperative pathological findings, for example, the American Joint Committee on Cancer (AJCC) staging systems [14], and the prognostic model by Raoof et al [15]. Preoperative predictors are missing in these systems to predict the prognosis of patients after hepatectomy in the preoperative period.

Imaging is of vital importance in the diagnosis and staging of ICC, and its qualitative description of ICC may hold prognostic value, as the correlation between imaging features and clinicopathological features of ICC has been recognized. For instance, the study by Asayama et al. [16] discovered that the degree of tumor enhancement in the delayed CT phase correlated well with the fibrous base mass; Koh et al. [17] demonstrated that tumor signal intensity in the hepatobiliary phase (HBP) of MRI indicated the fibrous base mass; Kim et al. [18] concluded that IMCC exhibiting diffuse hyperenhancement in the hepatic arterial phase (HAP) showed fewer central stromal and necrotic areas, a wider range of cellular areas, and a higher frequency of bile duct cell components during pathological evaluation; Nanashima et al. [19] suggested that increased CT attenuation was associated with ICC tumor vascularity; Ariizumi et al. [20] found that IMCC with marked enhancement within the tumor on arterial CT scans showed a favorable surgical outcome due to its less invasive histopathologic characteristics in patients with IMCC. Thus, prognostic assessment of ICC patients based on preoperative images is feasible. However, there are some controversies among different studies regarding the prognostic significance of specific or certain imaging features, e.g., Kim et al. concluded that patients with ICC presenting diffuse hyperenhancement had a better event-free survival (EFS) compared to hypoenhancement on HAP but had no effect on overall survival (OS) [18]. By contrast, a study by Teraoku et al. demonstrated an advantage of hyperenhancement compared to hypoenhancement solely in terms of OS, with no significance for EFS [21]. While a study noted [22] that the heterogeneity between rim-enhancement and hyperenhancement had a meaningful impact on patient survival, another recent study [23] argued that its heterogeneity had no differences in prognostic impact. Further, the study by Rhee et al. considered that tumor margin status was with no effect on OS [24], while Jiang et al. suggested that clear tumor margins were associated with better OS [25]. Hence, the aim of this study was to retrospectively assess the prognostic value of preoperative imaging features of CT and MRI based on a single-center base. Also, to comprehensively assess the association of different imaging features with clinical outcomes of EFS and OS, we performed a literature-based meta-analysis of ICC studies, stratified by imaging modality.

## Materials and methods

### Patients

Patients with ICC who underwent curative-intent resection at the First Affiliated Hospital of Nanjing Medical University (Nanjing, China) from 2010 to 2019 were identified. Inclusion criteria: (a) patients with postoperative pathological diagnosis of ICC; (b) CT or MRI examination within 3 months before surgery. Patients with incomplete clinicopathological data, missing follow-up data, preoperative treatment for ICC, combined hepatocellular carcinoma or other malignancies were excluded. Ultimately, 204 patients were included in the study population, divided into a CT group (161 patients) and an MRI group (43 patients) based on preoperative imaging modalities. The study was approved by the Institutional Review Board and complied with the Declaration of Helsinki. Given that the data were retrospective and anonymous, patient consent was waived. The following features were retrospectively analyzed, including clinical features (gender, age, carcinoembryonic antigen (CEA), carbohydrate antigen 19-9(CA 19-9)); pathological features (tumor necrosis); imaging features(tumor size, number of tumors, tumor margin status, tumor location, cirrhosis, vascular invasion, lymph node metastasis, bile duct dilatation, enhancement pattern, diffusion-weighted images (DWI) pattern, HBP signal intensity (SI) pattern,enhancing capsule, delayed enhancement). According to the clinical practice guidelines of the European Society of Medical Oncology, patients were monitored for CEA, CA 19-9, and lung and abdominal imaging every 3 months for the first 2 years after surgery and every 6 months thereafter. OS and EFS were defined as the date from the date of surgery to the date of death and the date of first tumor recurrence/metastasis, respectively. We included disease-free, recurrence-free, and event-free survival in the definition of EFS.

### Extraction and definition of image data

All CT and MRI images were evaluated by two radiologists (Q.X. and M.L., with 22 and 5 years of experience in liver imaging, respectively) in consensus. Both reviewers were aware of the diagnosis of ICC but were blinded to all other clinical-pathological findings. The imaging features included tumor size, tumor multiplicity, vascular invasion, lymph node metastasis, cirrhosis, the enhancement pattern of HAP, bile duct invasion, tumor margin status, tumor location, DWI diffusion restriction [26], peritumoral enhancement in HAP, HBP SI pattern, enhancing capsule, delayed enhancement. The definition of each imaging finding is described in Supplementary Materials.

### Search strategy

The search and study review procedures followed PRISMA guidelines. We attempted to include full-text articles published to date on the prognostic value of preoperative imaging features for OS and/or EFS in patients with ICC. References for this systematic review and meta-analysis were identified through searches of PubMed, the Cochrane Library, Web of Science, and EMBASE from inception to June 2022. We developed a search strategy for PubMed using disease-specific subject terms for databases, combined with text terms for imaging features and prognostic concepts. Details are shown in the Supplementary Materials. The search strategy was customized for each database. And manually searched the reference lists of all retrieved articles and previous systematic evaluations.

### Study selection

The following criteria for eligibility among studies were set before collecting the articles: (1) observational prognostic studies with a follow-up period longer than 6 months; (2) participants were diagnosed with ICC by postoperative pathology; (3) prognostic factors were CT or MRI reported preoperative imaging features, including one or more of the following: tumor enhancement pattern in HAP, bile duct invasion, tumor number, lymph node metastasis, tumor margin status, tumor site, DWI diffusion restricted area, peri-tumor enhancement in HAP, tumor necrosis, and tumor SI of HBP. (4) Endpoints are OS or EFS; (5) Risk point estimates are reported as hazard ratios (HRs) with 95% confidence interval (CI) unadjusted or adjusted. (6) when several articles were published by the same authors or group, the newest or most informative single article was selected.

Exclusion criteria were the following: (1) no information on OS or EFS; (2) duplicate or irrelevant articles; (3) letters to the editor and/or commentary, reviews, articles published in a book, or papers published in a non-English language (4) non-human study; (5) studies with inappropriate data for meta-analysis, such as incomplete or inconsistent data.

### Data extraction

Two researchers (D.W.S and Z.G.X) searched the articles and extracted the data independently. Discrepancies between the two investigators were resolved by reference to the original article as well as discussion, and any further disagreements were arbitrated by the senior investigator (G.W.J). We extracted the following information from publications: author, year of publication, country, patient number, imaging modality, imaging features, form of comparison, definition of disease, follow-up time, survival data (HRs and 95%CI), *P* value, K value.

We used a sequential approach when extracting unadjusted hazard ratios (HRs): (1) if HRs were available in the original text, we used the HRs and CI provided in the original text; (2) if the original text provided Kaplan-Meier (KM) curves for different sets of image features, we used Engauge Digitizer (version 12.1) software to extract the coordinates of the points on the curves to reconstruct the survival results using Tierney's method and calculate the HRs and CI [27]. (3) for articles that provided survival rates, standard deviations, and study numbers at fixed time points (3 or 5 years), the HRs of the studies and their CI were estimated assuming an exponential distribution of the time distribution of events [28].

### Research quality assessment

The methodological quality of the studies was evaluated by the Newcastle-Ottawa Scale (NOS). A score of 0-9 was allocated to each study, and studies with NOS scores ≥6 were considered high quality.The quality of the study is proportional to the NOS score.

### Statistical analyses

Categorical variables were represented as numbers and proportions. Categorical variables were compared using the χ2 test. Survival curves were generated using the Kaplan-Meier method and compared using the log-rank test. Cox regression analysis was used to analyze the relationship between variables and survival in the CT and MRI groups separately; HRs with 95% CI were calculated. Variables with p<0.05 in univariate analysis were included in multivariate analysis to identify the independent prognostic factors. In the meta-analysis, a priori lists of imaging characteristics were designed to identify predictor variables for survival after hepatectomy in patients with ICC, using evidence-based implicit knowledge. To quantify the predictive effect of these variables on patients' postoperative prognosis, we extracted HRs obtained from the studied Cox proportional hazard models, both unadjusted as well as adjusted. If there were two or more, unduplicated cohorts of studies exploring predictive variables, the HRs provided by the study were pooled in the meta-analysis. We performed separate meta-analyses of unadjusted HRs and adjusted HRs to explore the most precise prognostic significance of imaging features. We used HRs with 95% CI for OS and EFS, and the combined results are shown as forest plots. CIs were considered significant if they did not cross the line of no difference. The I^2^ statistic and Q test were performed to assess the effect of heterogeneity between studies on the results of the meta-analysis. If I^2^ > 50% or Q-test *P* < 0.1 was considered to have severe heterogeneity, a random-effects model was selected; otherwise, a fixed-effects model was used. To explore potential sources of heterogeneity and to assess the impact of subgroup factors on the combined results, subgroup analyses were performed when the literature included in the meta-analysis for imaging characteristics stratified by imaging modalities, disease definitions, or HRs acquisition modalities. Sensitivity analyses assessed the reliability of studies by omitting one study at a time and examining the effect of each study on the combined results. Visual inspection of funnel plots as well as Egger's and Begg's tests were carried out to assess publication bias. If publication bias was present, the stability of the results was further assessed using the trim-and-fill method. A two-sided *p*-value < 0.05 was deemed significant. Time to postoperative survival and time to disease recurrence reported in each study were converted to months. When tumor enhancement patterns were trichotomized, a net meta-analysis was employed to quantify the significance of different enhancement patterns on OS and EFS. The prognostic impact of various enhancement patterns was analyzed simultaneously by combining all the direct and indirect evidence. Natural log transformations of HRs were used, and their 95% CI were utilized to estimate standard errors. To include all comparisons within the same framework, we used hyperenhancement as the reference group in all studies and obtained HRs and 95%CI between rim-enhancement, hypoenhancement, and the reference group in the studies; we used a fixed-effects network meta-analysis model and assessed inconsistencies between the prognostic efficacy of enhancement modalities using the I^2^ statistic. We show network plots for all pairwise enhancement pattern comparisons in the network. Markov Chain Monte Carlo (MCMC) iterations were performed, and the trajectories, posterior distribution densities, and diagnostic convergence plots were constructed to test whether the number of iterations was sufficient for convergence and to ensure the stability of the results. We obtained HRs as the mean, 95% CI as the 2.5^th^ to 97.5^th^ percentiles and produced league tables. A ranked bar chart was used to rank the prognostic efficacy of the enhancement patterns and to derive the enhancement pattern with the best prognosis. All analyses were performed using SPSS (version 26.0), Stata SE (version 16.0), and R (version 4.2.1) with the gemtc package, network package, and coda package.

## Results

### Patient characteristics

A total of 204 patients undergoing partial hepatectomy were screened and recruited into this study, with 105 (51.47%) patients over 60 years of age and 86 (42.15%) females, 161 patients (78.9%) in the CT group and 43 patients (21.1%) in the MRI group. The baseline characteristics of the study cohort patients are shown in Table 1.

**Table 1:** Baseline characteristics of the study cohorts

Variables	CT group(*n* = 161)	MR group(*n* = 43)	*p* value
**Clinical characteristics**			
Gender (%)			
Male	92(57.14)	26(60.47)	0.695
Female	69(42.86)	17(39.53)	
Age(> 60 years,%)			
Yes	85(52.80)	20(46.51)	0.464
No	76(47.20)	23(53.49)	
History of biliary stones (%)			
Yes	19(11.80)	5(11.63)	0.975
No	142(88.20)	38(88.37)	
Hepatitis B virus infection (%)			
Yes	37(22.98)	12(27.90)	0.502
No	124(77.02)	31(72.10)	
CA19-9(> 37U/ml,%)			
Yes	94(58.39)	22(51.16)	0.396
No	67(41.61)	21(48.84)	
CEA(> 5 ng/ml,%)			
Yes	59(36.65)	12(27.90)	0.285
No	102(63.35)	31(72.10)	
**Pathologic characteristics**			
Peripheral tissue invasion (%)			
Yes	11(6.80)	1(2.30)	0.265
No	150(93.20)	42(97.70)	
Satellite nodules (%)			
Yes	31(19.25)	19(44.19)	0.001
No	130(80.75)	24(55.81)	
Vascular invasion (%)			
Yes	76(47.19)	7(16.28)	< 0.001
No	85(52.81)	36(83.72)	
Neural invasion (%)			
Yes	44(27.33)	3(7.00)	0.005
No	117(72.67)	40(93.00)	
R0 resection (%)			
Yes	137(85.09)	30(69.77)	0.021
No	24(14.91)	13(30.23)	
Lymph node metastasis (%)			
Yes	45(27.95)	7(16.28)	0.119
No	116(72.05)	36(83.72)	
Tumor necrosis (%)			
Yes	29(18.01)	8(18.60)	0.929
No	132(81.99)	35(81.40)	
**Imaging features**			
Tumor size(> 5 cm,%)			
Yes	90(55.90)	19(44.19)	0.171
No	71(44.10)	24(55.81)	
Tumor multiplicity (%)			
Yes	38(23.60)	7(16.28)	0.304
No	123(76.40)	36(83.72)	
Tumor boundary (%)			
Infiltrative	85(52.80)	13(30.23)	0.009
Distinct	76(47.20)	30(69.77)	
Tumor location (%)			
Peripheral	127(78.88)	40(93.00)	0.033
Perihilar	34(21.12)	3(7.00)	
Vascular invasion (%)			
Yes	55(34.16)	6(13.95)	0.009
No	104(65.84)	37(86.05)	
Lymph node metastasis (%)			
Yes	86(53.42)	7(16.28)	< 0.001
No	75(46.58)	36(83.72)	
Liver cirrhosis (%)			
Yes	23(14.29)	4(9.30)	0.392
No	138(85.71)	39(90.70)	
Bile duct invasion (%)			
Yes	87(54.04)	10(23.26)	< 0.001
No	74(45.96)	33(76.74)	
Arterial phase enhancement pattern (%)			< 0.001
Hyperenhancement	33(20.49)	16(37.22)	
Hypoenhancement	68(42.24)	12(27.90)	
Rim enhancement	60(37.27)	15(34.88)	
HBP SI pattern (%)	NA		NA
Intermediate		10(23.26)	
Hypointense		33(76.74)	
DWI pattern (%)	NA		NA
Diffusion restricted area < 1/3		13(30.23)	
Diffusion restricted area > 1/3		30(69.77)	
Enhancing capsule (%)			0.001
Yes	55(34.16)	4(9.30)	
No	106(65.84)	39(90.70)	

### Prognostic factors for ICC

We sought to explore independent prognostic factors in the original cohort using Cox regression models (Tables 2 and 3). A multivariate analysis showed that in the CT group, tumor multiplicity (HRs=2.213 95%CI 1.385-3.537, *p*=0.001), infiltration margin HRs=(2.565 95%CI 1.633-4.029, *p*<0.001), lymph node metastasis (HRs=2.975 95%CI 1.903-4.65, *p*<0.001), hypoenhancement (HRs=5.318 95%CI 2.564-11.027, *p*<0.001), rim-enhancement (HRs=2.926 95%CI 1.459-5.866, *p*=0.002), enhancing capsule (HRs=1.806 95%CI 1.005-3.039, *p*=0.025), high CEA levels (HRs=1.761 95%CI 1.160-2.674, *p*=0.008), and tumor necrosis (HRs=1.741 95%CI 1.083-2.798, *p*=0.022) were associated with poorer OS; tumor multiplicity (HRs=1.591 95%CI 1.04-2.435, *p*=0.032), tumor infiltrative margins (HRs=2.252 95%CI 1.478-3.432, *p*<0.001), lymph node metastasis (HRs=1.691 95%CI 1.129-2.531, *p*=0.011), hypoenhancement (HRs=4.274 95%CI 2.214-8.251, *p*<0.001), rim-enhancement (HRs=3.521 95%CI 1.848-6.708, *p*<0.001) tumor necrosis (HRs=1.598 95%CI 1.013-2.522, *p*=0.044) were associated with poorer EFS. In the MRI group, tumor multiplicity (HRs=6.524 95%CI 2.108-20.193, *p*=0.001), hypoenhancement (HRs=6.024 95%CI 1.605-22.613, *p*=0.008) were associated with poorer OS; tumor multiplicity (HRs=2.708 95%CI 1.017-7.208, *p*=0.046), hypoenhancement (HRs=4.971 95%CI 1.535-16.091, *p*=0.027) was associated with poorer EFS.

**Table 2:** Univariate and multivariate analysis results for overall survival and event-free survival; CT group

**Variables**	Overall Survival				Event-free Survival			
	Univariate analysis		Multivariable analysis		Univariate analysis		Multivariable analysis	
	Hazard ratio(95% confidence interval)	*P* value	Hazard ratio(95% confidence interval)	*P* value	Hazard ratio(95% confidence interval)	*P* value	Hazard ratio(95% confidence interval)	*P* value
**Clinicopathological features**								
Sex (female)	0.777(0.538–1.124)	0.180			0.881(0.624–1.244)	0.473		
Age(> 60)	1.084(0.755–1.557)	0.662			0.872(0.62–1.226)	0.430		
CEA	2.155(1.491–3.114)	< 0.001	1.761(1.160–2.674)	0.008	1.691(1.192–2.399)	0.003	1.252(0.841–1.863)	0.268
CA199	1.634(1.121–2.383)	0.011	1.078(0.697–1.668)	0.735	1.691(1.188–2.407)	0.004	0.825(0.558–1.220)	0.335
Tumor necrosis	1.721(1.100–2.693)	0.017	1.741(1.083–2.798)	0.022	1.769(1.154–2.712)	0.009	1.598(1.013–2.522)	0.044
**Imaging features**								
Size (> 5 cm)	2.715(1.485–3.186)	< 0.001	1.152(0.746–1.777)	0.524	2.488(1.738–3.563)	< 0.001	1.457(0.980–2.166)	0.063
Tumour multiplicity	2.432(1.615–3.660)	< 0.001	2.213(1.385–3.537)	0.001	2.190(1.480–3.241)	< 0.001	1.591(1.040–2.435)	0.032
Lesion contour (infiltrative)	3.096(2.099–4.565)	< 0.001	2.565(1.633–4.029)	< 0.001	2.745(1.915–3.935)	< 0.001	2.252(1.478–3.432)	< 0.001
Tumor location (perihilar)	1.150(0.748–1.768)	0.526			0.985(0.652–1.487)	0.943		
Cirrhosis	0.709(0.410–1.224)	0.217			0.700(0.415–1.180)	0.181		
Vascular invasion	1.775(1.223–2.576)	0.003	1.016(0.653–1.580)	0.945	1.387(0.977–1.969)	0.068		
Lymph node metastasis	3.274(2.212–4.845)	< 0.001	2.975(1.903–4.650)	< 0.001	2.566(1.792–3.676)	< 0.001	1.691(1.129–2.531)	0.011
Bile duct invasion	1.576(1.089–2.282)	0.016	1.186(0.757–1.860)	0.457	1.217(0.863–1.715)	0.262		
Arterial enhancement pattern		< 0.001		< 0.001		< 0.001		< 0.001
Hypoenhancement(vs. hyper)	7.776(4.072–14.847)	< 0.001	5.318(2.564–11.027)	< 0.001	7.018(3.824–12.879)	< 0.001	4.274(2.214–8.251)	< 0.001
Rim-enhancement(vs. hyper)	3.820(2.006–7.247)	< 0.001	2.926(1.459–5.866)	0.002	5.010(2.752–9.123)	< 0.001	3.521(1.848–6.708)	< 0.001
Enhancing capsule	0.627(0.420–0.936)	0.022	1.806(1.005–3.039)	0.025	0.631(0.435–0.917)	0.016	1.213(0.755–1.948)	0.425
Delayed enhancement	0.658(0.424–1.021)	0.062			1.021(0.691–1.508)	0.919

**Table 3:** Univariate and multivariate analysis results for overall survival and event-free survival; MRI group

**Variables**	Overall Survival				Event-free Survival			
	Univariate analysis		Multivariable analysis		Univariate analysis		Multivariable analysis	
	Hazard ratio(95% confidence interval)	*P* value	Hazard ratio(95% confidence interval)	*P* value	Hazard ratio(95% confidence interval)	*P* value	Hazard ratio(95% confidence interval)	*P* value
**Clinicopathological features**								
Sex (female)	0.369(0.134–1.011)	0.052			0.545(0.234–1.265)	0.158		
Age(> 60)	1.347(0.572–3.172)	0.496			1.555(0.707–3.416)	0.272		
CEA	2.084(0.861–5.044)	0.103			1.773(0.776–4.052)	0.174		
CA199	2.841(1.141–7.074)	0.025	1.633(0.619–4.311)	0.322	2.917(1.253–6.789)	0.013	1.589(0.625–4.035)	0.331
Tumor necrosis	1.998(0.729–5.482)	0.179			1.382(0.516–3.699)	0.520		
**Imaging features**								
Size (> 5 cm)	2.298(0.963–5.483)	0.061			1.900(0.861–4.195)	0.112		
Tumour multiplicity	8.762(2.94–26.094)	< 0.001	6.524(2.108–20.193)	0.001	4.241(1.675–10.734)	0.002	2.708(1.017–7.208)	0.046
Lesion contour (infiltrative)	1.365(0.550–3.390)	0.502			1.143(0.492–2.658)	0.755		
Tumor location (perihilar)	0.705(0.095–5.261)	0.733			1.019(0.235–4.410)	0.980		
Cirrhosis	1.740(0.511–5.928)	0.376			2.500(0.837–7.469)	0.101		
Vascular invasion	2.046(0.687–6.095)	0.198			2.371(0.884–6.356)	0.086		
Lymph node metastasis	2.376(0.863–6.541)	0.094			2.216(0.846–5.344)	0.109		
Bile duct invasion	1.100(0.402–3.004)	0.853			1.808(0.775–4.216)	0.171		
Arterial enhancement pattern		0.018		0.026		0.012		0.027
Hypoenhancement(vs. hyper)	6.644(1.787–24.697)	0.005	6.024(1.605–22.613)	0.008	5.746(1.809–18.249)	0.003	4.971(1.535–16.091)	0.007
Rim-enhancement(vs. hyper)	4.582(1.237–16.978)	0.023	3.240(0.839–12.501)	0.088	4.042(1.262–12.938)	0.019	2.986(0.879–10.152)	0.080
DWI pattern (< 1/3 vs. > 1/3)	0.439(0.125–1.543)	0.199			0.398(0.112–1.416)	0.155		
HBP SI pattern (intermediate vs. hypointense)	1.480(0.477–4.592)	0.498			1.382(0.445–4.294)	0.576		
Enhancing capsule	1.435(0.333–6.177)	0.628			1.045(0.245–4.453)	0.952		
Delayed enhancement	1.098(0.443–2.722)	0.840			1.564(0.691–3.542)	0.284		

### Literature search results and study characteristics

**Fig. 1:**
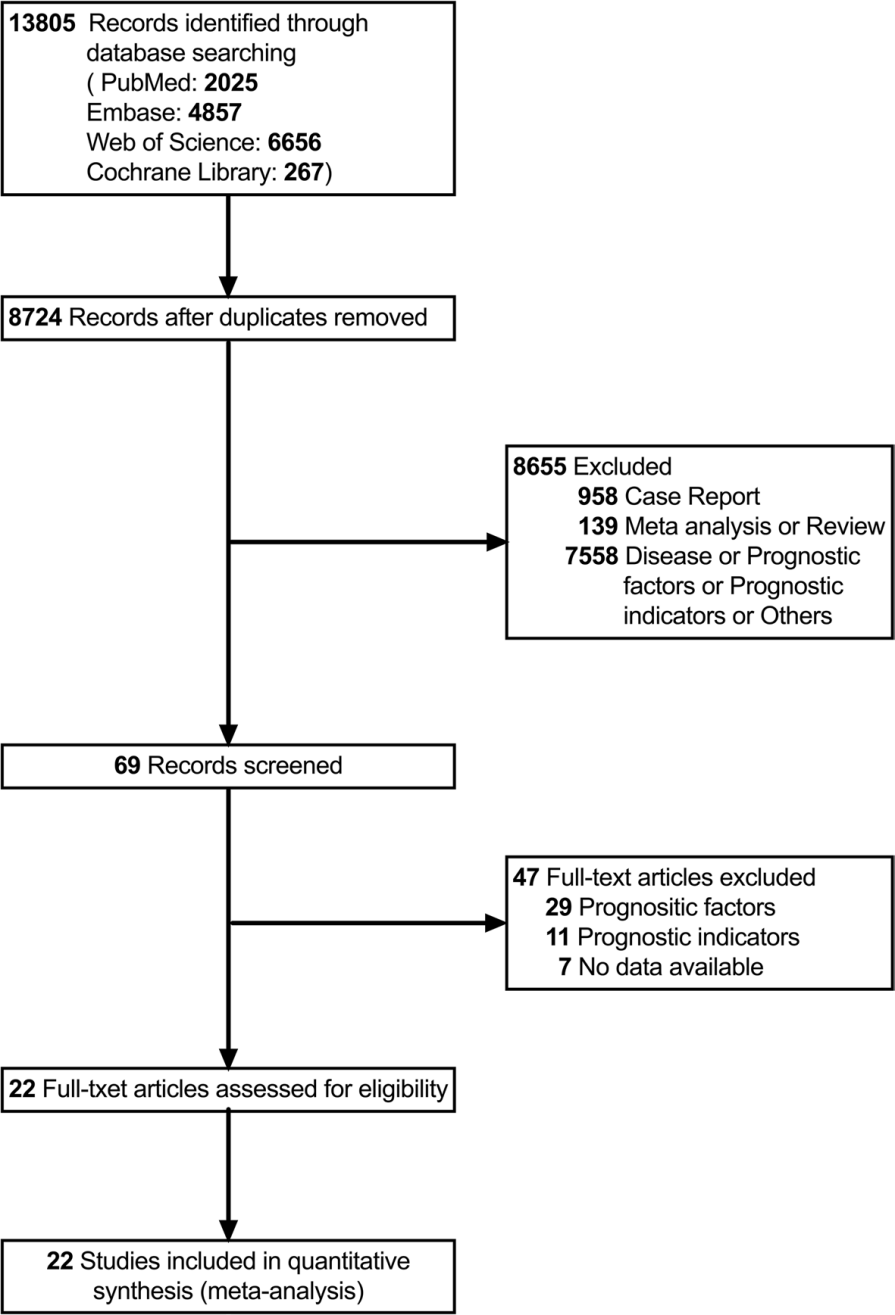
Literature search diagram

We initially identified 13,805 articles and screened their titles and abstracts (Fig. 1). Duplicates and irrelevant articles were excluded, leaving 69 to be further screened. We read the full text carefully while 47 studies that did not meet the inclusion requirements were excluded. Twenty-two articles matched the inclusion criteria [17–26, 29–40] and were therefore integrated into our meta-analysis, which explored a total of 8 prognostic imaging features for ICC patients. Twelve of these articles were included in a meta-analysis of multivariable HRs to quantitatively analyze the prognostic value of 3 imaging features. For all studies included in the meta-analysis, study quality was assessed according to NOS, with a median of 7 (range, 7-8). Tables 4 and 5 summarizes the characteristics of these studies. Including this study, a total of 21 articles probed OS [17–26, 31–40] and 16 articles probed EFS [17, 18, 21–23, 26, 29–32, 35–40] in patients with ICC.

**Table 4:** Predictor studies eligible for pooled analysis about adjusted HRs

Study ID	Country	Imaging examination method	Definition of disease	Trail Group	Control Group	Follow-up years	Primary endpoints	HR estimate	HR	95%CI	*P* value	NOS score	K value
				NO.of Hypoenhancement at arterial phase	NO.of Hyperenhancement at arterial phase								
Zhang 2020 [34]	China	MRI	ICC	40	28	4.7	OS	paper	1.878	1.063–3.320	0.030	8	0.83
Teraoku 2020 [21]	Japan	CT	IMCC	32	8	7.0	OS	paper	6.680	1.160–128.300	0.030	7	NA
Kim 2011 [18]	Korea	CT	IMCC	49	15	5.5	EFS	paper	3.011	1.252–7.238	0.014	7	NA
Fujita 2017 [29]	Japan	CT	IMCC	13	34	11.0	EFS	paper	5.853	2.069–17.399	< 0.001	8	NA
Ariizumi 2011 [20]	Japan	CT	IMCC	109	25	15.0	OS	paper	2.740	NA	0.045	8	NA
				NO. of Infiltrative tumor margin	NO. of absence								
Kim 2019 [31]	Korea	MRI	IMCC	NA	NA	8.0	EFS	paper	2.130	0.890–5.110	0.090	8	NA
Jiang 2011 [25]	China	CT or MRI	ICC	151	193	10.0	OS	paper	2.234	1.659–3.009	< 0.001	8	NA
Park 2021 [30]	Korea	CT	IMCC	27	206	4.8	EFS	paper	1.630	1.030–2.590	0.040	7	0.99
This study	China	CT	ICC	85	76	15.0	OS	paper	2.565	1.633–4.029	< 0.001	8	NA
							EFS	paper	2.252	1.478–3.432	< 0.001		
Rhee 2022 [24]	Korea	MRI	IMCC	32	176	8.0	OS	paper	NA	NA	NA	8	NA
				NO. of Bile duct invasion	NO. of absence								
Kim 2019 [31]	Korea	MRI	IMCC	33	21	8.0	OS	paper	2.040	0.770–5.40	0.151	8	NA
							EFS	paper	2.170	0.860–5.460	0.101		
Rhee 2022 [24]	Korea	MRI	IMCC	116	92	8.0	OS	paper	1.886	1.271–2.799	0.002	8	NA
This study	China	CT	ICC	87	74	15.0	OS	paper	1.186	0.757–1.860	0.457	8	NA
Min 2019 [22]	Korea	MRI	IMCC	54	80	8.0	OS	paper	NA	NA	NA	8	0.75–0.95
							EFS	paper	NA	NA	NA		
Jin 2022 [40]	China	MRI	IMCC	69	162	7.8	OS	paper	NA	NA	NA	8	NA
							EFS	paper	NA	NA	NA		
Park 2021 [30]	Korea	CT	IMCC	112	121	4.8	EFS	paper	NA	NA	NA	7	0.99
				NO. of Necrosis sign	NO. of absence								
Aherne 2018 [35]	USA	CT	ICC	25	41	8.0	OS	paper	3.180	1.290–7.860	0.012	7	NA
							EFS	paper	1.950	1.070–3.540	0.060		NA
Min 2019 [22]	Korea	MRI	IMCC	77	57	8.0	OS	paper	NA	NA	NA	8	0.75–0.95
							EFS	paper	NA	NA	NA		
Jin 2022 [40]	China	MRI	IMCC	145	86	7.8	OS	paper	NA	NA	NA	8	NA
							EFS	paper	NA	NA	NA		NA
				NO. of perihilar tumor location	NO. of peripheral tumor location								
Jiang 2011 [25]	China	CT or MRI	ICC	37	307	10.0	OS	paper	1.162	0.711–1.899	0.549	8	NA
Jin 2022 [40]	China	MRI	IMCC	102	129	7.8	OS	paper	NA	NA	NA	8	NA
							EFS	paper	NA	NA	NA		
Rhee 2022 [24]	Korea	MRI	IMCC	66	142	8.0	OS	paper	NA	NA	NA	8	NA
				NO. of The area of DWI Diffusion restriction accounted for the tumor area < 1/3	NO. of The area of DWI Diffusion restriction accounted for the tumor area > 1/3								
Promsorn 2022 [33]	Thailand	MRI	IMCC	29	44	5.0	OS	paper	2.039	1.180–3.535	0.011	8	NA
Lee 2016 [26]	Korea	MRI	IMCC	43	48	5.0	OS	paper	2.660	NA	0.024	8	NA
				NO.of Intermediate group	NO.of Hypointense group								
Koh 2016 [17]	Korea	MRI	IMCC	21	18	5.0	OS	paper	NA	NA	NA	8	NA
							EFS	paper	5.000	1.629–20.352	0.012		
Min 2019 [22]	Korea	MRI	IMCC	8	126	8.0	OS	paper	NA	NA	NA	8	0.75–0.95
							EFS	paper	NA	NA	NA		
				NO. of Peritumoral arterial hyperenhancement	NO. of absence								
Min 2019 [22]	Korea	MRI	IMCC	39	95	8.0	OS	paper	NA	NA	NA	8	0.75–0.95
							EFS	paper	NA	NA	NA		
Rhee 2022 [24]	Korea	MRI	IMCC	116	92	8.0	OS	paper	NA	NA	NA	8	NA

**Table 5:** Predictor studies eligible for net Meta-analysis about adjusted HRs

**Study ID**	**Country**	**Imaging examination method**	**Definition of disease**	**NO.of Hypoenhancement at arterial phase**	**NO.of Peripheral enhancement**	**NO.of Hyperenhancement at arterial phase**	**Form of comparison**	**Follow-up years**	**Primary endpoints**	**HR estimate**	**HR**	**95%CI**	***P*** value	**NOS score**	**K value**
Min 2019 [22]	Korea	MRI	IMCC	33	81	20	**Hypo:Hyper**	8	OS	paper	41.00	5.00–312.00	< 0.010	8	NA
							**Peri:Hyper**				11.00	2.00–85.00	0.020		
							**Hypo:Hyper**		EFS		3.50	1.40–8.60	< 0.010		
							**Peri:Hyper**				1.70	0.70–3.90	0.230		
Hyeong 2022 [37]	Korea	CT	ICC	60	56	23	**Hypo:Hyper**	15	EFS	paper	3.89	1.70–8.91	0.001	8	NA
							**Peri:Hyper**				6.24	2.67–14.59	< 0.001		
Panettieri 2021 [23]	USA	CT	ICC	10	29	17	**Hypo:Hyper**	5	OS	paper	5.33	1.52–19.90	0.009	7	NA
							**Peri:Hyper**				1.65	0.67–4.09	0.280		
							**Hypo:Hyper**		EFS		3.22	1.08–9.56	0.036		
							**Peri:Hyper**				1.75	0.75–4.08	0.192		
This study	China	CT	ICC	68	60	33	**Hypo:Hyper**	15	OS	paper	5.32	2.56–11.03	< 0.001	8	NA
							**Peri:Hyper**				2.93	1.46–5.87	0.002		
							**Hypo:Hyper**		EFS		4.27	2.21–8.25	< 0.001		
							**Peri:Hyper**				3.52	1.85–6.71	< 0.001		
		MRI		12	15	16	**Hypo:Hyper**		OS		5.14	1.32–19.97	0.018		
							**Peri:Hyper**				3.08	0.80–11.84	0.101		
							**Hypo:Hyper**		EFS		3.94	1.12–13.89	0.033		
							**Peri:Hyper**				2.63	0.75–9.18	0.129		
Jin 2022 [40]	China	MRI	IMCC	56	142	33	**Hypo:Hyper**	8	OS	paper	NA	NA	NA	8	NA
							**Peri:Hyper**				NA	NA			
							**Hypo:Hyper**		EFS		NA	NA			
							**Peri:Hyper**				NA	NA			
Park 2021 [30]	Korea	CT	IMCC	100	111	22	**Hypo:Hyper**	10	EFS	paper	NA	NA	NA	7	0.99
							**Peri:Hyper**				NA	NA			

### Meta-analysis of OS

**Fig. 2:**
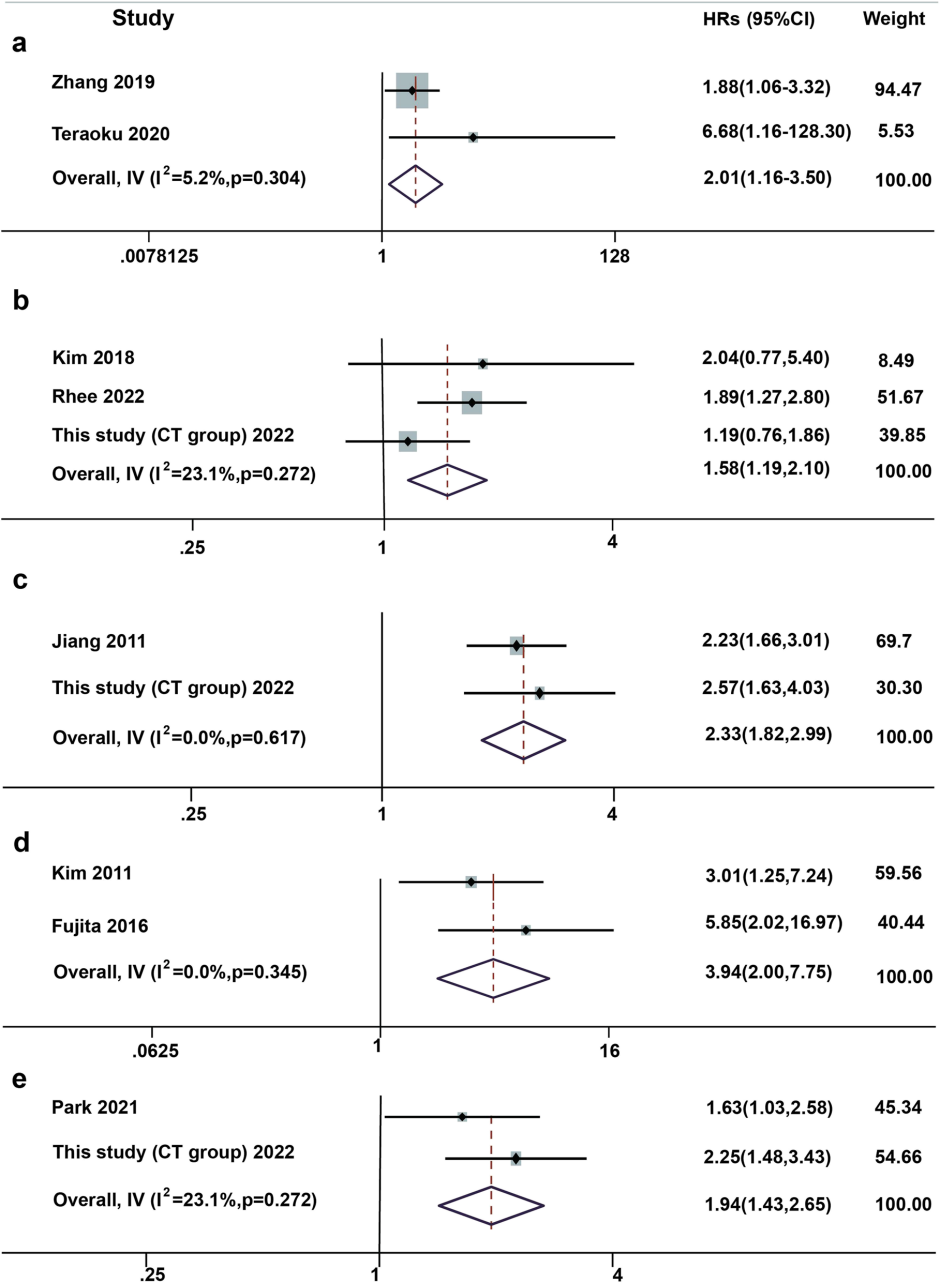
Forest plots and pooled effect estimates of predictors of overall survival and event-free survival. **a** Forest plot of enhancement pattern as a predictor of OS. **b** Forest plot of bile duct invision as a predictor of OS. **c** Forest plot of tumor margin status as a predictor of OS. **d** Forest plot of enhancement pattern as a predictor of EFS. **e** Forest plot of tumor margin status as a predictor of EFS. HR, hazard ratio; CI, confidence interval; OS, overall survival; EFS, event-free survival

Pooled results from two studies exploring arterial phase enhancement patterns showed an association with prognosis (HRs=2.01, 95% CI 1.16-3.50) (Fig. 2 a), with results indicating that low tumor enhancement significantly reduced patient survival relative to high enhancement. Two studies investigating the effect of bile duct invasion on patient survival also showed an association (HRs=1.58, 95% CI 1.19-2.10) (Fig. 2 b), with bile duct invasion leading to poorer survival. Exploration of tumor margin status showed a significant predictive effect of infiltrative tumor margin on poor survival (HRs=2.33,95%CI 1.82-2.99) (Fig. 2 c). Some studies were eligible for inclusion in the meta-analysis, but their HRs and 95% CI could not be combined due to differences in reference group selection [20], but similarly indicated that hypoenhancement of tumors was linked to poorer patient survival.

### Meta-analysis for EFS

A pooling of arterial phase enhancement patterns with patient recurrence rates showed that tumor hypoenhancement resulted in shorter EFS (HRs=3.94,95%CI 2.00-7.75) (Fig. 2d). Similarly, infiltrative tumor margins were associated with poorer EFS (HRs=1.94, 95% CI 1.43-2.65) (Fig. 2e). The results from meta-analysis of univariate HRs are summarized in Table S3 (Appendix). As only two studies were included in the meta-analysis, it was clearly inappropriate to perform subgroup analysis for enhancement pattern, tumor margin status, and DWI diffusion restriction area, so we conducted subgroup analysis only when pooling this predictor for bile duct invasion, and the results showed no effect of different imaging modalities or disease definitions on the pooled results. The results from meta-analysis of subgroups are detailed in the Supplementary Materials.

**Fig. 3:**
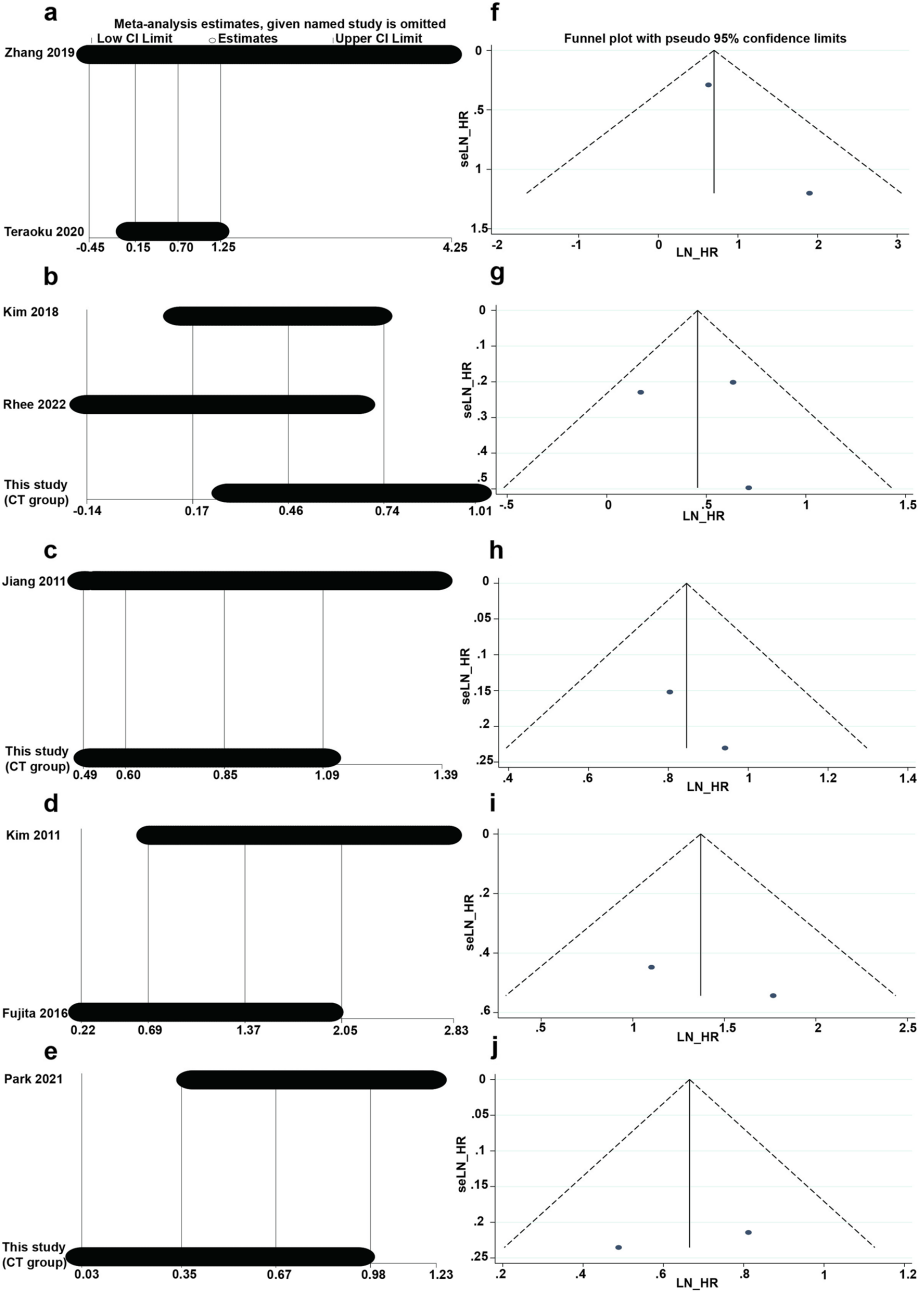
Results of influence analysis and funnel plots of predictors of overall survival and event-free survival. **a** Influence analysis of enhancement pattern as a predictor of OS. **b** Influence analysis of bile duct invasion as a predictor of OS. **c** Influence analysis of tumor margin status as a predictor of OS. **d** Influence analysis of enhancement pattern as a predictor of EFS. **e** Influence analysis of tumor margin status as a predictor of EFS. **f** Funnel plot of enhancement pattern as a predictor of OS. **g** Funnel plot of bile duct invasion as a predictor of OS. **h** Funnel plot of tumor margin status as a predictor of OS. **i** Funnel plot of enhancement pattern as a predictor of EFS. **j** Funnel plot of tumor margin status as a predictor of EFS. HR, hazard ratio; OS, overall survival; EFS, event-free survival

To verify the stability of this study, sensitivity analysis was undertaken by removing one study at a time. The results indicated that the removal of any one study had little effect on the combined results, indicating that the current results are reliable. As shown in Fig. 3 a-e, each point represents an independent study for the specified association, and visual inspection of the funnel plot (Fig. 3 f-j) and review of Egger's test *P* values (Table 5) did not indicate evidence of publication bias between articles.

### Results of net meta-analysis

**Fig. 4:**
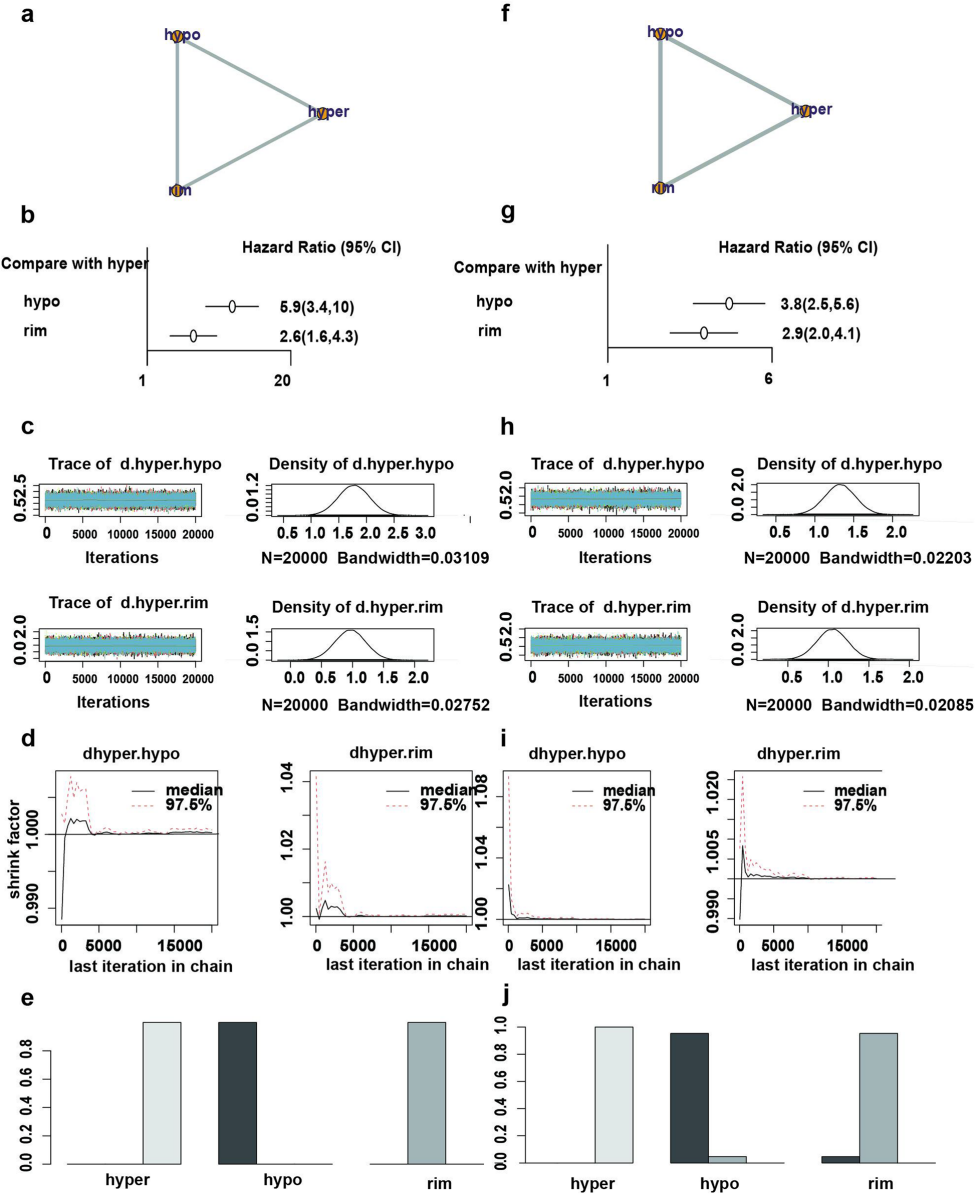
Net meta-analysis of enhancement pattern as a predictor of overall survival and event-free survival. **a** network plot of enhancement pattern as a predictor of OS. **b** Forest plot of enhancement pattern as a predictor of OS. **c** Trajectory plot and posterior distribution density plot of enhancement pattern as a predictor of OS. **d** Diagnostic convergence plot of enhancement pattern as a predictor of OS. **e** Ranked histogram of enhancement pattern as a predictor of OS. **f** network plot of enhancement pattern as a predictor of EFS. **g** Forest plot of enhancement pattern as a predictor of EFS. **h** Trajectory plot and posterior distribution density plot of enhancement pattern as a predictor of EFS. **i** Diagnostic convergence plot of enhancement pattern as a predictor of EFS. **j** Ranked histogram of enhancement pattern as a predictor of EFS. CI, confidence interval; hyper, hyperenhancement; rim, rim-enhancement; hypo, hypoenhancement

A total of 4 studies were screened into the net meta-analysis of triple enhancement patterns and OS, including 394 patients, and the meta-results showed that the comparative relationship between different enhancement patterns is illustrated in the net relationship plot (Fig. 4, a), where the size of the dot represents the number of people included in the study and the width of the line segment represents the count of included studies with hypoenhancement (HRs=5.90, 95% CI 3.40-10.00) versus rim-enhancement (HRs=2.60, 95% CI 1.60-4.30) (Table 6) resulted in worse OS with I^2^=8%. The combined results from the forest plot (Fig. 4 b), ranked probability, ranked histogram (Fig. 4 e), and league table (Fig. S1) showed that OS time was sequentially decreased in rim-enhanced and hypoenhanced ICC patients compared with hyperenhanced ICC patients, and that hypoenhancement was more likely to be associated with worse OS when compared with hyperenhanced ICC in terms of probability science relative to rim-enhancement.

**Table 6:** Combined results of adjusted HRs

Imaging features	Subgroup	No. of studies	No. of patients	Forms of comparison	Primary endpoints	Overall/Event-Free Survival			Heterogeneity		*P*>|t|
						HRs	95%CI		I²(%)	PQ	
Arterial phase enhancement pattern (2 Categories)	NA	2	108	Hypo/Hyper	OS	2.01	(1.16	3.50)	5.2	0.304	NA
	NA	2	111	Hypo/Hyper	EFS	3.94	(2.00	7.75)	0.0	0.345	NA
Invasion of bile duct	CT/ICC	1	161	Presence/Absence	OS	1.19	(0.76	1.86)	0.0	NA	0.889
	MRI/IMCC	2	262			1.91	(1.32	2.75)	0.0	0.884	
	Subtotal	3	423			1.58	(1.19	2.10)	23.1	0.272	
Infiltrating tumor margin	NA	2	405	Presence/Absence	OS	2.33	(1.82	2.99)	0.0	0.617	NA
	NA	2	394		EFS	1.94	(1.43	2.65)	3.1	0.310	NA
Arterial phase enhancement pattern (3 Categories)	NA	4	394	Hypo/Hyper	OS	5.90	(3.40	10.00)	8.0	NA	NA
				Rim/Hyper		2.60	(1.60	4.30)			
		5	533	Hypo/Hyper	EFS	3.80	(2.50	5.60)	22.0		NA
				Rim/Hyper		2.90	(2.00	4.10)			

**Fig. 5:**
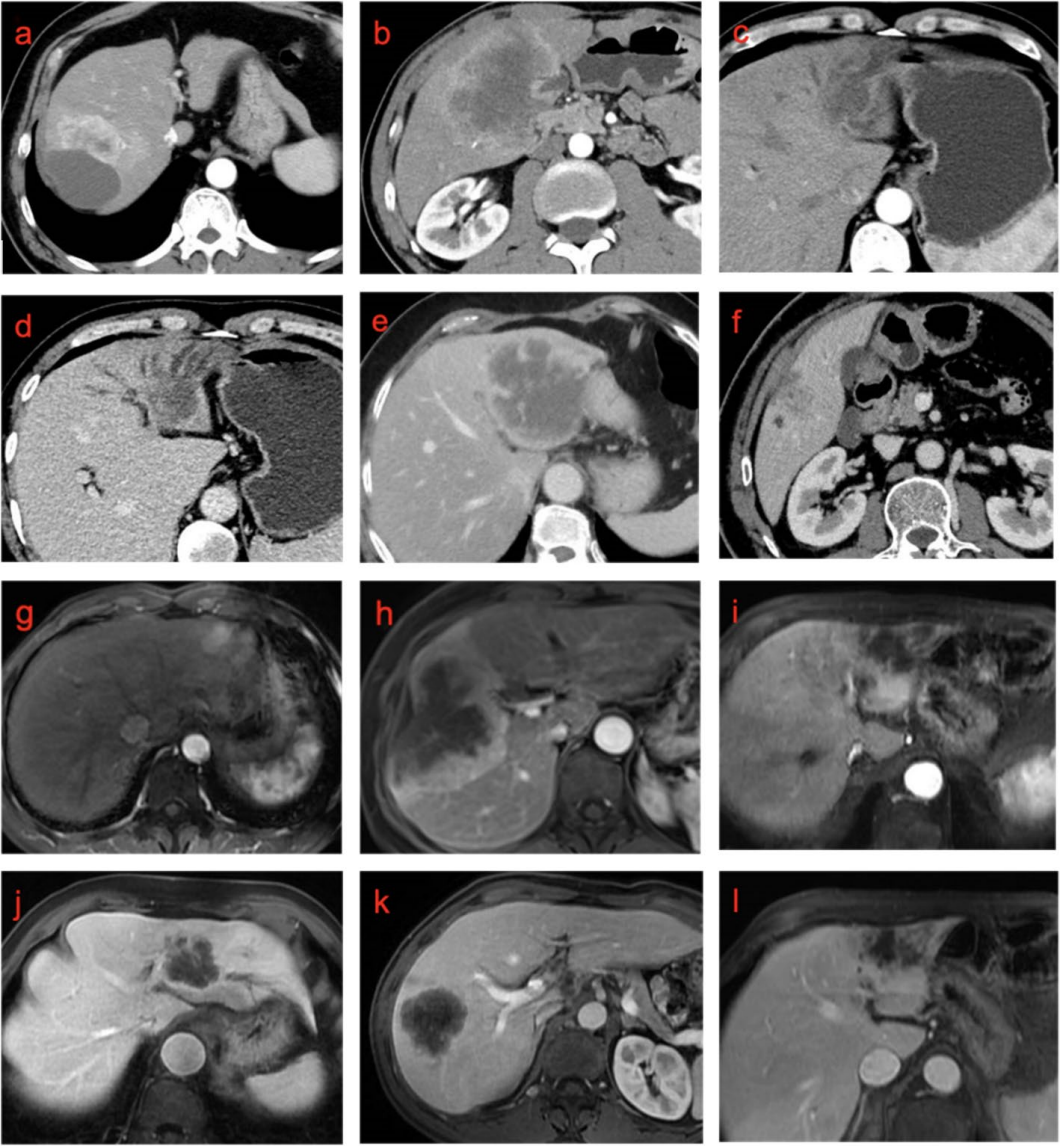
Examples of imaging features of patients with ICC. **a** Hyperenhanced ICC in CT images. **b** Rim-enhanced ICC in CT images. **c** Hypoenhanced ICC in CT images. **d** Bile duct invasion in CT images. **e** Clear tumor margin in CT images. **f** Infiltrative tumor margin in CT images. **g** Hyperenhanced ICC in MRI images. **h** Rim-enhanced ICC in MRI images. **i** Hypoenhanced ICC in MRI images. **j** Bile duct invasion in MRI images. **k** Clear tumor margin in MRI images. **l** Infiltrative tumor margin in MRI images

After screening, five studies that met the inclusion criteria, including 533 patients, were included in the net meta-analysis of triple enhancement patterns and EFS. The meta-results revealed that the comparative relationship between different enhancement patterns is shown in the net relationship plot (Fig. 4 f), with hypoenhancement (HRs=3.80, 95% CI 2.50-5.60) versus rim-enhancement pattern (HRs=2.90, 95% CI 2.00-4.10) (Table 6) resulted in shorter EFS with I^2^=22%. The combined results from forest plot (Fig. 5 b), ranked probability, ranked histogram (Fig. 4 j), and league table (Fig. S2) showed that EFS was sequentially reduced in rim-enhanced and hypoenhanced ICC patients compared with hyperenhanced ICC patients, while hypoenhancement was probabilistically more likely to be associated with poorer EFS compared with hyperenhanced ICC relative to rim-enhancement.

Based on the trajectory plots, the posterior distribution density plots (Fig. 4 c, h), the diagnostic convergence plots (Fig. 4 d, i), and the results of the scale reduction factors (all 1), it can be seen that constructed net meta-analysis model converges well.

## Discussion

Increasingly, studies have demonstrated the relevance between imaging features and tumor pathology [16–20], and multiple studies have compared the prognostic and predictive role of different imaging features on postoperative OS or EFS in patients with ICC [17–26, 29–40], but these studies were single-center with limited sample size and no meta-analysis was performed. We used a combination of single-center data and meta-analysis to analyze the prognostic efficacy of imaging features. A comprehensive retrospective analysis of the prognostic imaging features for ICC patients was performed.

The inconsistent results between CT and MRI groups may be associated with differences in sample size as well as discrepancy in observation of different tissues between CT and MRI. This discrepancy also exists between different studies. We did our best to collect previous studies on prognostic imaging features for meta-analysis. Tumor multiplicity, extrahepatic organ invasion, and lymph node metastasis are well-known factors associated with poor prognosis in ICC and are also included in the AJCC staging system and other postoperative prognostic systems [14, 25, 41–44], and therefore were not included in the meta-analysis. The meta-analysis included eight variables: enhancement pattern in HAP, bile duct invasion, tumor margin status, DWI diffusion restriction area, HBP SI pattern, tumor site, necrosis sign, and peritumor enhancement in HAP. However, due to the small number of adjusted HRs values available, the multivariate HRs meta-analysis only included enhancement pattern in HAP, bile duct invasion, and tumor margin status. The results of the univariate HRs meta-analysis are shown in the additional material. The results of multivariate HRs meta-analysis showed that HAP enhancement pattern and tumor margin status were associated with OS and EFS in ICC patients; bile duct invasion was only associated with postoperative OS. The heterogeneity of the combined results was low, and the statistical results were of high quality.

The arterial phase enhancement pattern is classified in a variable manner, with hyperenhancement and rim-enhancement patterns defined according to more than 50% and 10%-50% of the tumor enhancement area, respectively [29], or using 70% as the cut-off point [22] ,or without a predefined threshold value [30]. Meanwhile, some studies included only hyper- and hypoenhancment patterns [18–20, 29, 34, 38]. In order to cover more literatures, no area size requirement was made for enhancement patterns, and a net meta-analysis was performed. The results of a separate meta-analysis of studies with dichotomous enhancement patterns showed that hypoenhancement was associated with poorer OS and EFS. Through the net meta-analysis, we can perform the prognostic analysis of three enhancement patterns (hyperenhancement,rim-enhancement,hypoenhancement)simultaneously after aggregating the direct prognostic results and indirect prognostic results between any two enhancement patterns. Net Meta-analysis showed that the prognosis of ICC with hyperenhancement, rim-enhancement, and hypoenhancement decreased gradually. Figure 5 a,b,c,g,h,i show representative images of different enhancement patterns for ICCs. Clinicians can make prognostic judgments according to the CT and MRI features of ICCs. It has been demonstrated that areas of tumor hypoenhancement are linked to poor differentiation, necrosis, and degree of fibrosis. Conversely, highly differentiated adenocarcinomas showed a greater degree of tumor enhancement [45]. It has also been advocated that enhancement pattern heterogeneity is associated with background liver injury [46–49]. Patients with hyper-enhancing ICC have a higher prevalence of chronic liver disease; hyperenhanced-ICC represents an early stage before the acquisition of advanced malignant features [50, 51]. Additionally, Teraoku et al. [21] suggested that hypodense foci in the center of ICC lesions were significantly associated with HIF-1 expression. Overexpression of HIF-1a in several cancers is relevant to angiogenesis, cell proliferation and survival, and accelerated tumor malignancy [52]. Studies not involved in the meta-analysis also confirmed the better prognosis of highly enhancing ICC [20, 24]; nevertheless, there were also studies that did not include enhancement pattern as a prognostic factor, probably because the prognostic impact of imaging features was statistically underestimated in a small number of subjects, considering the relatively high prognostic power of pathological features [31, 32]. Compared with unresectable cases, hyperenhancement pattern is more frequent in resectable ICCs, thus, enhancement patterns in HAP may be used for treatment decisions, including the feasibility of surgery and the choice of neoadjuvant therapy [23].

Pooled findings demonstrate that bile duct invasion is associated with poorer OS. Figure 5 d,j show the specific manifestations of bile duct invasion in CT and MRI images. ICC can be subdivided into small duct and large duct types reflecting the origin of the tumor. According to the definition of bile duct invasion, the bile duct visible to the naked eye may be the site of origin of ICC, which implies that it is an imaging presentation of large duct type ICC with worse prognosis [53, 54]. Subgroup analysis showed heterogeneity stemming from the imaging modality, with better agreement between studies using MRI as preoperative images, which may be due to the excellent soft tissue contrast of MRI. Summary results showed that infiltrative tumor margins can be a prognostic factor for ICC. However, several studies [22, 30, 40] showed no prognostic significance for bile duct invasion but were not included in the meta-analysis because they did not meet the inclusion criteria. Also in combination with the prognostic results of our data, the prognostic significance of bile duct invasion needs to be further examined. Figure 5 e,f,k,l illustrate the appearance of clear and unclear tumor margins of ICC patients in CT and MRI images. This finding was supported by other studies but was not included in the analysis due to the presence of duplicate cohorts [31]. Infiltrative margins are a good indicator of tumor micro-infiltration [55]. Consequently, tumor margin status may serve as an important indication of the extent of liver resection, guiding clinicians in making treatment plans and improving patient survival after surgery.

For reasons involving replicate cohorts, experimental design, inability to extract and combine HRs, the remaining variables in our review were not integrated into the meta-analysis, but conclusions can be drawn from the qualitative analysis. Several studies [24, 25, 33] have confirmed that tumor location has no effect on prognosis. Two studies [26, 33] found that the diffusion restricted area of DWI tumors correlated with survival, and ICC with diffusion restricted area less than 1/3 had more advanced TNM staging, more common lymph node metastases, and more abundant interstitial connective tissue hyperplasia [26]. The important role of DWI images was also illustrated in a study by Lewis et al [56]. Studies on SI pattern analysis of HBP [17, 22] showed that different SI patterns had no effect on patient survival, and the combination of univariate HRs showed that it led to a higher recurrence rate. Two studies [22, 24] involving peri-tumor enhancement in HAP did not conclude a meaningful effect on prognosis, and the combination of univariate HRs was found to be associated with postoperative recurrent metastasis. According to some studies [38], internal tumor necrosis signs are a marker of poor prognosis in patients with ICC, and our CT group also found that pathological tumor necrosis causes adverse prognosis, but more studies are needed to support this. Rhee et al. [24] investigated that periductal tumor invasion is associated with poor OS; Park et al. [30] suggested that it leads to a shorter EFS. In the 7th edition of the AJCC TNM staging system, once periductal tumor infiltration is detected, it is directly classified as T4, but it is not included in the 8th edition of the AJCC TNM staging due to unclear prognostic significance [14]. In addition to the above, there are still some imaging features without clear evidence of prognostic significance. To improve the postoperative survival of ICC patients in clinical practice, we expect to see more studies on prognostic imaging features of ICC.

There are some limitations of this paper. The studies at our center and the included studies were retrospective and limited in number. The criteria regarding the definition of imaging features were not uniform among studies at different centers, so the pooled results are somewhat flawed. Although studies from the same registry within overlapping time frames may not contain the same cohort, there were insufficient methods for exclusion, which may have resulted in incomplete data. Non-English texts were also excluded from our analysis. Finally, the likelihood of false positive results increased with the analysis of many predictor variables.

## Conclusion

In conclusion, arterial enhancement pattern, and tumor margin status at preoperative CT/MRI imaging are associated with both recurrence and survival of ICC patients following resection that can be incorporated into the prognostic system to guide individualized treatment in future clinical practice.

## Supplementary Information

**Additional file 1.**

**Additional file 2.**

**Additional file 3.**

**Additional file 4.**

**Additional file 5:** **Table S1.** Predictor studies eligible for pooled analysis about unadjusted HRs

**Additional file 6:** **Table S2.** Predictor studies eligible for net Meta-analysis about unadjusted HRs

**Additional file 7:** **Table S3.** Combined results of unadjusted HRs

## Data Availability

All data generated or analyzed during this study are included in this published article.

## Abbreviations

CA 19–9: Carbohydrate antigen 19–9
CEA: Carcinoembryonic antigen
CI: Confidence interval
CT: Computed tomography
DWI: Diffusion-weighted images
EFS: Event-free survival
HAP: Hepatic arterial phase
HBP: Hepatobiliary phase
HRs: Hazard ratios
ICC: Intrahepatic cholangiocarcinoma
IMCC: Intrahepatic mass-forming cholangiocarcinoma
MRI: Magnetic resonance imaging
OS: Overall survival
SI: Signal intensity
