# Supplementary material for: Imaging features based on CT and MRI for predicting prognosis of patients with intrahepatic cholangiocarcinoma: a single-center study and meta-analysis
Source: Cancer Imaging. 2023 Jun 7;23:56. doi: 10.1186/s40644-023-00576-5 (PMC10245452; doi:10.1186/s40644-023-00576-5)
Supplement: Supplementary file 1 — Additional file 1. [file 40644_2023_576_MOESM1_ESM.docx]

The evaluation criteria for CT and MR image feature extraction are as follows. (a) Tumor size is defined as the longest diameter of the lesion in the axial scan. (b) Tumor multiplicity is defined as the presence of satellite foci or intrahepatic metastases. (c) Vascular invasion is defined as tortuosity, encirclement, occlusion, or tumor thrombosis of large intrahepatic vessels. (d) Lymph node metastasis is defined as enlarged lymph nodes with a short diameter >1 cm and internal necrosis or abnormal morphology. (e) Cirrhosis is defined as an irregular or atrophic liver with signs of ascites or portal hypertension. (f) The enhancement pattern of HAP is divided into two categories, one is the dichotomous classification of enhancement pattern, defined as diffuse hyperenhancement and diffuse hypoenhancement; the other is the trichotomous classification of enhancement pattern, with the addition of rim-enhancement. (g) Bile duct invasion is defined as tumor encapsulation of large segments of intrahepatic bile ducts and or dilatation of peritumoral bile ducts. (h) Tumor border status, clear tumor border is defined as a tumor with a clear border of adjacent liver parenchyma; an infiltrative tumor border is defined as a tumor with an unclear border of adjacent liver parenchyma. (i) Tumor location, the perihepatic location was defined as the main tumor occurred in the second or third branch of the intrahepatic bile duct, or the case was accompanied by a dilatation of the intrahepatic bile duct; perihepatic location was defined as the main tumor was judged to be in the peripheral liver. (j) DWI diffusion restriction, defined as the presence of high signal intensity on high b-value DWI (b-value = 800 s/mm2) and equal low signal intensity on the corresponding ADC map. The diffusion restricted areas were estimated qualitatively by visual assessment in axial MRI slices with the largest tumor axis and were divided into two groups (1) restricted areas equal to or greater than 1/3 of the tumor and (2) restricted areas less than 1/3 of the tumor [26]. (k) Peritumoral enhancement in the arterial phase, defined as partial crescentic or polygonal enhancement detectable outside the tumor margin in the arterial phase. (l) Hepatobiliary phase (HBP) tumor signal intensity, defined as a high signal or equisignal area exceeding 50% of the tumor area compared to the spleen in the intermediate signal group; and low signal group defined as a high signal or equisignal area below 50% of the tumor area compared to the spleen [17]. (m) Enhancing capsule, defined as uniform enhancement of the peripheral rim in the portal or delayed phase. (n) Delayed enhancement, defined as progressive enhancement in the venous or portal phase.

**Reference for supplementary methods**

[1] Koh J, Chung YE, Nahm JH, et al (2016) Intrahepatic mass-forming cholangiocarcinoma: prognostic value of preoperative gadoxetic acid-enhanced MRI. Eur Radiol 26:407–416.
