# Supplementary material for: Imaging features based on CT and MRI for predicting prognosis of patients with intrahepatic cholangiocarcinoma: a single-center study and meta-analysis
Source: Cancer Imaging. 2023 Jun 7;23:56. doi: 10.1186/s40644-023-00576-5 (PMC10245452; doi:10.1186/s40644-023-00576-5)
Supplement: Supplementary file 2 — Additional file 2. [file 40644_2023_576_MOESM2_ESM.docx]

**PubMed search formula**

((("Cholangiocarcinoma"[Mesh]) OR ((((((((((((((((Cholangiocarcinomas[Title/Abstract]) OR (Cholangiocellular Carcinoma[Title/Abstract])) OR (Intrahepatic mass-forming cholangiocarcinoma[Title/Abstract])) OR (Mass-forming intrahepatic cholangiocarcinoma[Title/Abstract])) OR (MF-ICCA[Title/Abstract])) OR (ICC[Title/Abstract])) OR (IMCC[Title/Abstract])) OR (Carcinoma, Cholangiocellular[Title/Abstract])) OR (Carcinomas, Cholangiocellular[Title/Abstract])) OR (Cholangiocellular Carcinomas[Title/Abstract])) OR (Intrahepatic Cholangiocarcinoma[Title/Abstract])) OR (Cholangiocarcinoma, Intrahepatic[Title/Abstract])) OR (Cholangiocarcinomas, Intrahepatic[Title/Abstract])) OR (Intrahepatic Cholangiocarcinomas[Title/Abstract])) OR (Resectable intrahepatic cholangiocarcinoma[Title/Abstract])) OR (Resectable[Title/Abstract] AND unresectable intrahepatic cholangiocarcinoma[Title/Abstract]))) AND (((((((((((((((((((((((((((((Diagnosed[Title/Abstract]) OR (Prognosis[Title/Abstract])) OR (Survival rate[Title/Abstract])) OR (Mortality[Title/Abstract])) OR (Death[Title/Abstract])) OR (Predictor[Title/Abstract])) OR (Predictors[Title/Abstract])) OR (Cohort*[Title/Abstract])) OR (Treatment outcome*[Title/Abstract])) OR (Recurrence[Title/Abstract])) OR (Prognostic marker*[Title/Abstract])) OR (Disease-free survival[Title/Abstract])) OR (Clinical outcomes[Title/Abstract])) OR (Prognosis model[Title/Abstract])) OR (Overall survival[Title/Abstract])) OR (Disease-free survival[Title/Abstract])) OR (Preoperative Prognostic Marker[Title/Abstract])) OR (Prognostic Factor*[Title/Abstract])) OR (Surgical Outcome*[Title/Abstract])) OR (clinicopathological finding*[Title/Abstract])) OR (Clinicopathologic Characteristic*[Title/Abstract])) OR (prognostic value[Title/Abstract])) OR (Prognostic impact[Title/Abstract])) OR ( postoperative prognosis prediction[Title/Abstract])) OR (prognostic prediction[Title/Abstract])) OR (Preoperative Prognostic Marker*[Title/Abstract])) OR (long-term survival[Title/Abstract])) OR (Prognostic*[Title/Abstract])) OR (clinicopathologic feature*[Title/Abstract]))) AND ((((((((((((((((((((((((((((((((((((((Tomography, X-Ray Computed[Title/Abstract]) OR (X-Ray Computed Tomography[Title/Abstract])) OR (Tomography, X-Ray Computerized[Title/Abstract])) OR (CT[Title/Abstract])) OR (Computed Tomography[Title/Abstract])) OR (Magnetic resonance imaging[Title/Abstract])) OR (Imaging, Magnetic Resonance[Title/Abstract])) OR (MRI[Title/Abstract])) OR (Imaging[Title/Abstract])) OR (image feature[Title/Abstract])) OR (Imaging findings[Title/Abstract])) OR (image character*[Title/Abstract])) OR (Diagnostic imaging[Title/Abstract])) OR (Image interpretation,computed-assisted[Title/Abstract])) OR (Enhanced imaging[Title/Abstract])) OR (Enhancement patterns[Title/Abstract])) OR (Hepatobiliary phase[Title/Abstract])) OR (Rim-enhancement[Title/Abstract])) OR (Diffusion weighted Imaging[Title/Abstract])) OR (Dynamic CT Enhancement[Title/Abstract])) OR (Delayed-Phase Dynamic CT Enhancement[Title/Abstract])) OR (gadoxetic acid-enhanced MRI[Title/Abstract])) OR (Arterial enhancement pattern[Title/Abstract])) OR (tumor vascularity on CT[Title/Abstract])) OR (arterial[Title/Abstract] AND delayed enhancement patterns[Title/Abstract])) OR (Gadoxetic acid enhanced magnetic resonance imaging[Title/Abstract])) OR (preoperative gadoxetic acid-enhanced MRI[Title/Abstract])) OR (gadoxetic acid-enhanced MRI[Title/Abstract])) OR (Pattern of Tumor Enhancement[Title/Abstract])) OR (tumor imaging enhancement[Title/Abstract])) OR (Enhancement on Arterial-Phase Computed Tomography[Title/Abstract])) OR (Arterial-Phase Computed Tomography[Title/Abstract])) OR (Arterial Enhancement Patterns at MRI[Title/Abstract])) OR (Arterial Enhancement Patterns*[Title/Abstract])) OR (Enhancement patterns in the arterial phase of dynamic hepatic CT[Title/Abstract])) OR (dynamic hepatic CT[Title/Abstract])) OR (magnetic resonance imaging-based[Title/Abstract])) OR (Computed tomography-based[Title/Abstract]))
