# Supplementary material for: Imaging features based on CT and MRI for predicting prognosis of patients with intrahepatic cholangiocarcinoma: a single-center study and meta-analysis
Source: Cancer Imaging. 2023 Jun 7;23:56. doi: 10.1186/s40644-023-00576-5 (PMC10245452; doi:10.1186/s40644-023-00576-5)
Supplement: Supplementary file 3 — Additional file 3. [file 40644_2023_576_MOESM3_ESM.pdf]

Log Hazard Ratio (95% CrI)

hyper	1.777 (1.225, 2.324)	0.9669 (0.4788, 1.448)
-1.777 (-2.324, -1.225)	hypo	-0.8109 (-1.254, -0.366)
-0.9669 (-1.448, -0.4788)	0.8109 (0.366, 1.254)	rim
