# Supplementary material for: Imaging features based on CT and MRI for predicting prognosis of patients with intrahepatic cholangiocarcinoma: a single-center study and meta-analysis
Source: Cancer Imaging. 2023 Jun 7;23:56. doi: 10.1186/s40644-023-00576-5 (PMC10245452; doi:10.1186/s40644-023-00576-5)
Supplement: Supplementary file 4 — Additional file 4. [file 40644_2023_576_MOESM4_ESM.pdf]

Log Hazard Ratio (95% CrI)

| | | |
|---|---|---|
| hyper | 1.324 (0.9305, 1.718) | 1.049 (0.6792, 1.42) |
| -1.324 (-1.718, -0.9305) | hypo | -0.2752 (-0.5954, 0.04696) |
| -1.049 (-1.42, -0.6792) | 0.2752 (-0.04696, 0.5954) | rim |
