# Supplementary material for: Imaging features based on CT and MRI for predicting prognosis of patients with intrahepatic cholangiocarcinoma: a single-center study and meta-analysis
Source: Cancer Imaging. 2023 Jun 7;23:56. doi: 10.1186/s40644-023-00576-5 (PMC10245452; doi:10.1186/s40644-023-00576-5)
Supplement: Supplementary file 5 — Additional file 5: Table S1. Predictor studies eligible for pooled analysis about unadjusted HRs [file 40644_2023_576_MOESM5_ESM.docx]

Table S1 Predictor studies eligible for pooled analysis about unadjusted HRs

| **Study ID** | **Country** | **Imaging examination method** | **Definition of disease** | **Trail Group** | **Control Group** | **Follow-up years** | **Primary endpoints** | **HR estimate** | **HR** | **95%CI** | **NOS score** | **K value** |
| --- | --- | --- | --- | --- | --- | --- | --- | --- | --- | --- | --- | --- |
|  |  |  |  | **NO.of Hypoenhancement at arterial phase** | **NO.of Hyperenhancement at arterial phase** |  |  |  |  |  |  |  |
| Zhang 2019 | China | MR | ICC | 40 | 28 | 4.7 | OS | paper | 1.946 | 1.114-3.399 | 8 | 0.832 |
| Nanashima 2013 | Japan | CT | ICC | 30 | 12 | 6 | OS | indirect | 2.48 | 1.1-5.58 | 7 | NA |
| Kim 2011 | Korea | CT | IMCC | 49 | 15 | 5.5 | OS | indirect | 1.5 | 0.69-3.26 | 7 | NA |
|  |  |  |  |  |  |  | EFS | indirect | 2.44 | 1.24-4.83 |  |  |
| Ariizumi 2011 | Japan | CT | IMCC | 109 | 25 | 15 | OS | indirect | 2.99 | 1.72-5.19 | 8 | NA |
| Teraoku 2020 | Japan | CT | IMCC | 32 | 8 | 7 | OS | indirect | 3.28 | 1.06-10.12 | 7 | NA |
|  |  |  |  |  |  |  | EFS | indirect | 1.85 | 0.76-4.48 |  |  |
| Mehmet 2016 | Japan | CT | ICC | 49 | 21 | 10 | OS | indirect | 2.01 | 1.02-3.96 | 7 | NA |
|  |  |  |  | **NO.of Intermediate group** | **NO.of Hypointense group** |  |  |  |  |  |  |  |
| Kim 2018 | Korea | MR | IMCC | NA | NA | 8 | OS | paper | 0.71 | 0.31-1.6 | 8 | NA |
|  |  |  |  |  |  |  | EFS | paper | 1.52 | 0.66-3.51 |  |  |
| Koh 2015 | Korea | MR | IMCC | 21 | 18 | 5 | OS | indirect | 3.07 | 1-9.49 | 8 | NA |
|  |  |  |  |  |  |  | EFS | indirect | 4.28 | 1.69-10.82 |  |  |
| This study | China | MR | ICC | **10** | **33** | 15 | OS | paper | 1.48 | 0.477-4.592 | 8 | NA |
|  |  |  |  |  |  |  | EFS | paper | 1.382 | 0.445-4.294 |  |  |
|  |  |  |  | **NO. of the area of DWI Diffusion restriction accounted for the tumor area ＜1/3** | **NO. of the area of DWI Diffusion restriction accounted for the tumor area ＞1/3** |  |  |  |  |  |  |  |
| Kim 2018 | Korea | MR | IMCC | NA | NA | 8 | OS | paper | 1.49 | 0.57-3.9 | 8 | NA |
|  |  |  |  |  |  |  | EFS | paper | 1.26 | 0.52-3.08 |  |  |
| Lee 2016 | Korea | MR | IMCC | 43 | 48 | 5 | OS | indirect | 2.59 | 1.47-4.57 | 8 | NA |
|  |  |  |  |  |  |  | EFS | indirect | 2.46 | 1.44-4.22 |  |  |
| Promsorn 2022 | Thailand | MR | IMCC | 29 | 44 | 5 | OS | indirect | 2.41 | 1.43-4.09 | 8 | NA |
| This study | China | MR | ICC | **13** | **30** | 15 | OS | paper | 0.439 | 0.125-1.543 | 8 | NA |
|  |  |  |  |  |  |  | EFS | paper | 0.398 | 0.112-1.416 |  |  |
|  |  |  |  | **NO. of Peritumoral arterial hyperenhancement** | **NO. of absence** |  |  |  |  |  |  |  |
| Kim 2018 | Korea | MR | IMCC | NA | NA | 8 | OS | paper | 1.88 | 0.64-5.53 | 8 | NA |
|  |  |  |  |  |  |  | EFS | paper | 1.71 | 0.64-1.54 |  |  |
| Min 2019 | Korea | MR | IMCC | 39 | 95 | 8 | OS | paper | 2.5 | 1.5-4.2 | 8 | 0.75-0.95 |
|  |  |  |  |  |  |  | EFS | paper | 2.2 | 1.4-3.4 |  |  |
| Park 2021 | Korea | CT | IMCC | 78 | 155 | 4.8 | EFS | paper | 1.32 | 0.94-1.85 | 7 | 0.99 |
| Rhee 2022 | Korea | MR | IMCC | 116 | 92 | 8 | OS | paper | 1.425 | 0.996-2.039 | 8 | NA |
| Li 2022 | China | CT | ICC | 47 | 209 | 7 | OS | paper | 1.327 | 0.964-1.826 | 8 | NA |
|  |  |  |  |  |  |  | EFS | paper | 1.181 | 0.864-1.614 |  |  |
|  |  |  |  | **NO. of Infiltrative tumor margin** | **NO. of absence** |  |  |  |  |  |  |  |
| Kim 2018 | Korea | MR | IMCC | 33 | 21 | 8 | OS | paper | 1.4 | 0.6-3.24 | 8 | NA |
|  |  |  |  |  |  |  | EFS | paper | 2.35 | 0.98-5.63 |  |  |
| Rhee 2022 | Korea | MR | IMCC | 32 | 176 | 8 | OS | paper | 1.601 | 1.03-2.49 | 8 | NA |
| Park 2021 | Korea | CT | IMCC | 27 | 206 | 4.8 | EFS | paper | 2.22 | 1.43-3.47 | 7 | 0.99 |
| Bartsch 2020 | Germany | CT or MR | ICC | 59 | 66 | 7 | OS | indirect | 1.39 | 0.94-2.05 | 8 | NA |
|  |  |  |  |  |  |  | EFS | indirect | 1.37 | 0.94-2 |  |  |
| Jiang 2011 | China | CT or MR | ICC | 151 | 193 | 10 | OS | indirect | 1.54 | 1.19-2 | 8 | NA |
| This study | China | CT | ICC | **85** | **76** |  | OS | paper | 3.096 | 2.099-4.565 | 8 |  |
|  |  |  |  |  |  |  | EFS |  | 2.745 | 1.915-3.935 |  |  |
|  |  | MR |  | **13** | **30** |  | OS |  | 1.365 | 0.550-3.390 |  |  |
|  |  |  |  |  |  |  | EFS |  | 1.143 | 0.492-2.658 |  |  |
|  |  |  |  | **NO. of perihilar tumor location** | **NO. of peripheral tumor location** |  |  |  |  |  |  |  |
| YAMASHITA 2016 | Japan | CT | ICC | 34 | 44 | 16 | OS | indirect | 2.4 | 1.35-4.3 | 7 | NA |
|  |  |  |  |  |  |  | EFS | indirect | 1.43 | 0.8-2.51 |  |  |
| Jin 2022 | China | MR | IMCC | 102 | 129 | 7.8 | OS | paper | 2.278 | 1.608-3.227 | 8 | NA |
|  |  |  |  |  |  |  | EFS | paper | 2.337 | 1.743-3.243 |  |  |
| Min 2019 | Korea | MR | IMCC | 22 | 112 | 8 | OS | paper | 1 | 0.5-1.9 | 8 | 0.75-0.95 |
|  |  |  |  |  |  |  | EFS | paper | 0.7 | 0.4-1.4 |  |  |
| Jiang 2011 | China | CT or MR | ICC | 37 | 307 | 10 | OS | indirect | 1.38 | 0.91-2.08 | 8 | NA |
| Bartsch 2020 | Germany | CT or MR | ICC | 49 | 76 | 7 | OS | indirect | 1.34 | 0.9-2 | 8 | NA |
|  |  |  |  |  |  |  | EFS | indirect | 1.2 | 0.82-1.75 |  |  |
| Rhee 2022 | Korea | MR | IMCC | 66 | 142 | 8 | OS | paper | 2.14 | 3.06 | 8 | NA |
| This study | China | CT | ICC | **34** | **127** |  | OS | paper | 1.15 | 0.748-1.768 | 8 |  |
|  |  |  |  |  |  |  | EFS |  | 0.985 | 0.652-1.487 |  |  |
|  |  | MR |  | **3** | **40** |  | OS |  | 0.705 | 0.095-5.261 |  |  |
|  |  |  |  |  |  |  | EFS |  | 1.019 | 0.235-4.410 |  |  |
|  |  |  |  | **NO. of Bile duct invasion** | **NO. of absence** |  |  |  |  |  |  |  |
| Kim 2018 | Korea | MR | IMCC | 33 | 21 | 8 | OS | paper | 2.49 | 0.98-6.35 | 8 | NA |
|  |  |  |  |  |  |  | EFS | paper | 2.35 | 0.98-5.62 |  |  |
| Rhee 2022 | Korea | MR | IMCC | 116 | 92 | 8 | OS | paper | 2.67 | 1.822-3.912 | 8 | NA |
| Park 2021 | Korea | CT | IMCC | 112 | 121 | 4.8 | EFS | paper | 2.11 | 1.52-2.93 | 7 | 0.99 |
| Min 2019 | Korea | MR | IMCC | 54 | 80 | 8 | OS | paper | 2.6 | 1.6-4.3 | 8 | 0.75-0.95 |
|  |  |  |  |  |  |  | EFS | paper | 1.8 | 1.2-2.8 |  |  |
| Jin 2022 | China | MR | IMCC | 69 | 162 | 7.8 | OS | paper | 1.629 | 1.14-2.328 | 8 | NA |
|  |  |  |  |  |  |  | EFS | paper | 1.441 | 1.043-1.99 |  |  |
| Li 2022 | China | CT | ICC | 104 | 152 | 7 | OS | paper | 1.232 | 0.955-1.59 | 8 | NA |
| This study | China | CT | ICC | **87** | **74** |  | OS | paper | 1.576 | 1.089-2.282 | 8 |  |
|  |  |  |  |  |  |  | EFS |  | 1.217 | 0.863-1.715 |  |  |
|  |  | MR |  | **10** | **33** |  | OS |  | 1.1 | 0.402-3.004 |  |  |
|  |  |  |  |  |  |  | EFS |  | 1.808 | 0.775-4.216 |  |  |
|  |  |  |  | **NO. of Necrosis sign** | **NO. of absence** |  |  |  |  |  |  |  |
| Aherne 2018 | USA | CT | ICC | 25 | 41 | 8 | OS | paper | 2.95 | 1.44-6.04 | 7 | NA |
|  |  |  |  |  |  |  | EFS | paper | 1.95 | 1.07-3.54 |  |  |
| Min 2019 | Korea | MR | IMCC | 77 | 57 | 8 | OS | paper | 6.1 | 2.9-12.9 | 8 | 0.75-0.95 |
|  |  |  |  |  |  |  | EFS | paper | 3.7 | 2.2-6.4 |  |  |
| Jin 2022 | China | MR | IMCC | 145 | 86 | 7.8 | OS | paper | 2.742 | 1.833-4.101 | 8 | NA |
|  |  |  |  |  |  |  | EFS | paper | 2.69 | 1.896-3.815 |  |  |
| Li 2022 | China | CT | ICC | 74 | 182 | 7 | OS | paper | 1.069 | 0.811-1.41 | 8 | NA |
|  |  |  |  |  |  |  | EFS | paper | 1.182 | 0.904-1.545 |  |  |
