# Supplementary material for: Imaging features based on CT and MRI for predicting prognosis of patients with intrahepatic cholangiocarcinoma: a single-center study and meta-analysis
Source: Cancer Imaging. 2023 Jun 7;23:56. doi: 10.1186/s40644-023-00576-5 (PMC10245452; doi:10.1186/s40644-023-00576-5)
Supplement: Supplementary file 6 — Additional file 6: Table S2. Predictor studies eligible for net Meta-analysis about unadjusted HRs [file 40644_2023_576_MOESM6_ESM.docx]

Table S2 Predictor studies eligible for net Meta-analysis about unadjusted HRs

| **Study ID** | **Country** | **Imaging examination method** | **Definition of disease** | **NO. of Hypoenhancement at arterial phase** | **NO. of Peripheral enhancement** | **NO. of Hyperenhancement at arterial phase** | **Form of comparison** | **Follow-up years** | **Primary endpoints** | **HR estimate** | **HR** | **95%CI** | **NOS score** | **K value** |
| --- | --- | --- | --- | --- | --- | --- | --- | --- | --- | --- | --- | --- | --- | --- |
| Fujita 2016 | Japan | CT | IMCC | 13 | 18 | 16 | **Hypo:Peri** | 11 | EFS | indirect | 4.2 | 1.78-9.89 | 8 | NA |
|  |  |  |  |  |  |  | **Hypo:Hyper** |  |  |  | 4.69 | 1.87-11.78 |  |  |
|  |  |  |  |  |  |  | **Peri:Hyper** |  |  |  | 1.38 | 0.48-3.96 |  |  |
| Hyeong 2021 | Korea | CT | ICC | 60 | 56 | 23 | **Hypo:Peri** | 15 | OS | indirect | 2.6 | 1.61-4.21 | 8 | NA |
|  |  |  |  |  |  |  | **Hypo:Hyper** |  |  |  | 3.07 | 1.16-8.15 |  |  |
|  |  |  |  |  |  |  | **Peri:Hyper** |  |  |  | 1.24 | 0.45-3.46 |  |  |
|  |  |  |  |  |  |  | **Hypo:Peri** |  | EFS |  | 1.87 | 1.27-2.75 |  |  |
|  |  |  |  |  |  |  | **Hypo:Hyper** |  |  |  | 6.656 | 3.112-14.236 |  |  |
|  |  |  |  |  |  |  | **Peri:Hyper** |  |  |  | 2.945 | 1.375-6.31 |  |  |
| Teraoku 2022 | Japan | CT | IMCC | 25 | 7 | 8 | **Hypo:Peri** | 7 | OS | indirect | 1.81 | 0.59-5.56 | 7 | NA |
|  |  |  |  |  |  |  | **Hypo:Hyper** |  |  |  | 3.55 | 1.13-11.18 |  |  |
|  |  |  |  |  |  |  | **Peri:Hyper** |  |  |  | 2.64 | 0.38-18.21 |  |  |
|  |  |  |  |  |  |  | **Hypo:Peri** |  | EFS |  | 0.56 | 0.18-1.72 |  |  |
|  |  |  |  |  |  |  | **Hypo:Hyper** |  |  |  | 1.91 | 0.79-4.63 |  |  |
|  |  |  |  |  |  |  | **Peri:Hyper** |  |  |  | 2.17 | 0.47-10.02 |  |  |
| Panettieri 2022 | USA | CT | ICC | 10 | 29 | 17 | **Hypo:Peri** | 5 | OS | indirect | 1.61 | 0.65-3.99 | 7 | NA |
|  |  |  |  |  |  |  | **Hypo:Hyper** |  |  |  | 3.21 | 1.05-9.78 |  |  |
|  |  |  |  |  |  |  | **Peri:Hyper** |  |  |  | 1.87 | 0.79-4.44 |  |  |
|  |  |  |  |  |  |  | **Hypo:Peri** |  | EFS |  | 1.46 | 0.66-3.22 |  |  |
|  |  |  |  |  |  |  | **Hypo:Hyper** |  |  |  | 3.21 | 1.21-8.52 |  |  |
|  |  |  |  |  |  |  | **Peri:Hyper** |  |  |  | 2.05 | 1-4.21 |  |  |
| Min 2019 | Korea | MRI | IMCC | 33 | 81 | 20 | **Hypo:Hyper** | 8 | OS | paper | 36 | 5-266 | 8 | NA |
|  |  |  |  |  |  |  | **Peri:Hyper** |  |  |  | 15 | 2-106 |  |  |
|  |  |  |  |  |  |  | **Hypo:Hyper** |  | EFS |  | 6.2 | 2.6-14.5 |  |  |
|  |  |  |  |  |  |  | **Peri:Hyper** |  |  |  | 2.8 | 1.3-6.2 |  |  |
| Jin 2022 | China | MRI | IMCC | 56 | 142 | 33 | **Hypo:Hyper** | 8 | OS | paper | 10.85 | 4.62-25.51 | 8 | NA |
|  |  |  |  |  |  |  | **Peri:Hyper** |  |  |  | 3.82 | 1.66-8.78 |  |  |
|  |  |  |  |  |  |  | **Hypo:Hyper** |  | EFS |  | 6.67 | 3.53-12.6 |  |  |
|  |  |  |  |  |  |  | **Peri:Hyper** |  |  |  | 2.78 | 1.53-5.06 |  |  |
| Park 2021 | Korea | CT | IMCC | 100 | 111 | 22 | **Hypo:Hyper** | 10 | EFS | paper | 2.38 | 1.25-4.51 | 7 | NA |
|  |  |  |  |  |  |  | **Peri:Hyper** |  |  |  | 2.06 | 1.09-3.91 |  |  |
| This study | China | CT | ICC | 68 | 60 | 33 | **Hypo:Hyper** |  | OS | paper | 7.776 | 4.072-14.847 | 8 | NA |
|  |  |  |  |  |  |  | **Peri:Hyper** |  |  |  | 3.82 | 2.006-7.247 |  |  |
|  |  |  |  |  |  |  | **Hypo:Hyper** |  | EFS |  | 7.018 | 3.824-12.879 |  |  |
|  |  |  |  |  |  |  | **Peri:Hyper** |  |  |  | 5.01 | 2.752-9.123 |  |  |
|  |  | MRI |  | 12 | 15 | 16 | **Hypo:Hyper** |  | OS |  | 6.644 | 1.787-24.697 |  |  |
|  |  |  |  |  |  |  | **Peri:Hyper** |  |  |  | 4.582 | 1.237-16.978 |  |  |
|  |  |  |  |  |  |  | **Hypo:Hyper** |  | EFS |  | 5.746 | 1.809-18.249 |  |  |
|  |  |  |  |  |  |  | **Peri:Hyper** |  |  |  | 4.042 | 1.262-12.938 |  |  |
