# Supplementary material for: Imaging features based on CT and MRI for predicting prognosis of patients with intrahepatic cholangiocarcinoma: a single-center study and meta-analysis
Source: Cancer Imaging. 2023 Jun 7;23:56. doi: 10.1186/s40644-023-00576-5 (PMC10245452; doi:10.1186/s40644-023-00576-5)
Supplement: Supplementary file 7 — Additional file 7: Table S3. Combined results of unadjusted HRs [file 40644_2023_576_MOESM7_ESM.docx]

| Imaging features | Subgroup | No. of studies | No. of patients | Forms of comparison | Primary endpoints | Overall/Event-Free Survival | | Heterogeneity | | P>\|t\| |
| --- | --- | --- | --- | --- | --- | --- | --- | --- | --- | --- |
|  |  |  |  |  |  | HRs | 95%CI | I²(%) | PQ |  |
| Arterial phase enhancement pattern(2 Categories) | CT | 5 | 350 | Hypo/Hyper | OS | 2.36 | 1.71-3.27 | 0.0 | 0.632 | 0.92 |
|  | MR | 1 | 68 |  |  | 1.95 | 1.11-3.41 | 0.0 | NA |  |
|  | ICC | 3 | 180 |  |  | 2.08 | 1.42-3.04 | 0.0 | 0.886 |  |
|  | IMCC | 3 | 238 |  |  | 2.48 | 1.63-3.77 | 12.6 | 0.319 |  |
|  | Indirect HRs | 5 | 350 |  |  | 2.36 | 1.71-3.27 | 0.0 | 0.632 |  |
|  | Direct HRs | 1 | 68 |  |  | 1.95 | 1.11-3.41 | 0.0 | NA |  |
|  | Subtotal | 6 | 418 |  |  | 2.25 | 1.70-2.98 | 0.0 | 0.714 |  |
|  | CT | 2 | 104 |  | EFS | 2.2 | 1.28-3.78 | 0.0 | 0.627 | NA |
|  | Subtotal | 2 | 104 |  |  | 2.2 | 1.28-3.78 | 0.0 | 0.627 |  |
| Peri-tumor enhancement in arterial phase | MR/IMCC | 3 | 396 | Presence/Absence | OS | 1.72 | 1.3-2.29 | 35.8 | 0.211 | 0.404 |
|  | CT/ICC | 1 | 256 |  |  | 1.33 | 0.96-1.83 | 0.0 | NA |  |
|  | Subtotal | 4 | 652 |  |  | 1.54 | 1.24-1.90 | 34.0 | 0.208 |  |
|  | CT/ICC | 2 | 489 |  | EFS | 1.24 | 0.99-1.56 | 0.0 | 0.636 | 0.016 |
|  | MR/IMCC | 1 | 134 |  |  | 2.20 | 1.41-3.43 | 0.0 | NA |  |
|  | Subtotal | 3 | 623 |  |  | 1.46 | 1.04-2.05 | 61.8 | 0.073 |  |
| Invasion of bile duct | CT | 2 | 417 | Presence/Absence | OS | 1.34 | 1.07-1.69 | 13.4 | 0.283 | 0.389 |
|  | MR | 5 | 670 |  |  | 2.11 | 1.58-2.81 | 32.1 | 0.207 |  |
|  | ICC | 3 | 460 |  |  | 1.32 | 1.08-1.62 | 0.0 | 0.525 |  |
|  | IMCC | 4 | 627 |  |  | 2.21 | 1.67-2.92 | 28.7 | 0.24 |  |
|  | Subtotal | 7 | 1087 |  |  | 1.78 | 1.36-2.34 | 62.1 | 0.015 |  |
|  | CT | 3 | 650 |  | EFS | 1.43 | 0.99-2.07 | 77.1 | 0.013 | 0.372 |
|  | MR | 3 | 408 |  |  | 1.58 | 1.24-2.02 | 0.0 | 0.679 |  |
|  | ICC | 3 | 460 |  |  | 1.21 | 0.99-1.47 | 0.0 | 0.621 |  |
|  | IMCC | 3 | 598 |  |  | 1.75 | 1.39-2.22 | 24.7 | 0.265 |  |
|  | Subtotal | 7 | 1058 |  |  | 1.49 | 1.21-1.85 | 51.3 | 0.068 |  |
| Infiltrating tumor margin | CT | 1 | 161 | Presence/Absence | OS | 3.10 | 2.10-4.57 | 0.0 | NA | 0.917 |
|  | MR | 3 | 305 |  |  | 1.52 | 1.06-2.18 | 0.0 | 0.931 |  |
|  | CT or MR | 2 | 469 |  |  | 1.49 | 1.20-1.85 | 0.0 | 0.668 |  |
|  | ICC | 4 | 673 |  |  | 1.79 | 1.20-2.66 | 72.3 | 0.013 |  |
|  | IMCC | 2 | 262 |  |  | 1.56 | 1.05-2.30 | 0.0 | 0.782 |  |
|  | Indirect HRs | 2 | 469 |  |  | 1.49 | 1.20-1.85 | 0.0 | 0.668 |  |
|  | Direct HRs | 4 | 456 |  |  | 1.91 | 1.22-3.00 | 57.4 | 0.071 |  |
|  | Subtotal | 6 | 925 |  |  | 1.72 | 1.30-2.28 | 55.4 | 0.047 |  |
|  | CT | 2 | 394 |  | EFS | 2.52 | 1.91-3.34 | 0.0 | 0.466 | 0.065 |
|  | MR | 1 | 43 |  |  | 1.14 | 0.49-2.66 | 0.0 | NA |  |
|  | CT or MR | 1 | 125 |  |  | 1.37 | 0.94-2.00 | 0.0 | NA |  |
|  | ICC | 3 | 329 |  |  | 1.72 | 0.98-3.02 | 75.8 | 0.016 |  |
|  | IMCC | 1 | 233 |  |  | 2.22 | 1.43-3.46 | 0.0 | NA |  |
|  | Indirect HRs | 1 | 125 |  |  | 1.37 | 0.94-2.00 | 0.0 | NA |  |
|  | Direct HRs | 3 | 437 |  |  | 2.19 | 1.49-3.23 | 44.1 | 0.167 |  |
|  | Subtotal | 4 | 562 |  |  | 1.87 | 1.26-2.77 | 65.5 | 0.034 |  |
| Tumor location | CT | 2 | 239 | Perihilar/Peripheral | OS | 1.62 | 0.79-3.32 | 75.0 | 0.046 | 0.322 |
|  | MR | 4 | 616 |  |  | 1.81 | 1.24-2.64 | 48.7 | 0.119 |  |
|  | CT or MR | 2 | 469 |  |  | 1.36 | 1.02-1.81 | 0.0 | 0.92 |  |
|  | ICC | 5 | 751 |  |  | 1.41 | 1.11-1.79 | 13.6 | 0.327 |  |
|  | IMCC | 3 | 573 |  |  | 1.87 | 1.27-2.75 | 58.5 | 0.09 |  |
|  | Indirect HRs | 3 | 547 |  |  | 1.55 | 1.13-2.14 | 32.9 | 0.225 |  |
|  | Direct HRs | 5 | 777 |  |  | 1.59 | 1.09-2.31 | 62.2 | 0.032 |  |
|  | Subtotal | 8 | 1324 |  |  | 1.60 | 1.26-2.04 | 50.9 | 0.047 |  |
|  | CT | 2 | 239 |  | EFS | 1.12 | 0.79-1.59 | 5.7 | 0.303 | 0.298 |
|  | MR | 3 | 408 |  |  | 1.26 | 0.48-3.31 | 83.8 | 0.002 |  |
|  | CT or MR | 1 | 125 |  |  | 1.20 | 0.82-1.75 | 0.0 | NA |  |
|  | ICC | 4 | 407 |  |  | 1.15 | 0.90-1.47 | 0.0 | 0.762 |  |
|  | IMCC | 2 | 365 |  |  | 1.33 | 0.40-4.40 | 91.5 | 0.001 |  |
|  | Indirect HRs | 2 | 203 |  |  | 1.27 | 0.92-1.74 | 0.0 | 0.619 |  |
|  | Direct HRs | 4 | 569 |  |  | 1.19 | 0.61-2.33 | 83.7 | 0.001 |  |
|  | Subtotal | 6 | 772 |  |  | 1.26 | 0.84-1.88 | 74.3 | 0.002 |  |
| DWI diffusion restricted area | Indirect HRs | 2 | 164 | ＜1/3 vs ＞1/3 | OS | 2.49 | 1.69-3.66 | 0.0 | 0.855 | 0.058 |
|  | Direct HRs | 2 | 97 |  |  | 0.87 | 0.26-2.85 | 56.4 | 0.13 |  |
|  | ICC | 1 | 43 |  |  | 0.44 | 0.12-1.54 | 0.0 | NA |  |
|  | IMCC | 3 | 218 |  |  | 2.32 | 1.62-3.32 | 0.0 | 0.163 |  |
|  | Subtotal | 4 | 261 |  |  | 1.74 | 0.97-3.13 | 58.4 | 0.065 |  |
|  | Indirect HRs | 1 | 91 |  | EFS | 2.46 | 1.44-4.21 | 0.0 | NA | 0.086 |
|  | Direct HRs | 2 | 97 |  |  | 0.78 | 0.25-2.37 | 53.0 | 0.145 |  |
|  | ICC | 1 | 43 |  |  | 0.4 | 0.11-1.42 | 0.0 | NA |  |
|  | IMCC | 2 | 145 |  |  | 1.94 | 1.04-3.63 | 37.2 | 0.207 |  |
|  | Subtotal | 3 | 188 |  |  | 1.23 | 0.48-3.20 | 72.5 | 0.026 |  |
| HBP SI pattern | Indirect HRs | 1 | 39 | Intermediate group/Hypointense group | OS | 3.07 | 1.00-9.45 | 0.0 | NA | 0.203 |
|  | Direct HRs | 2 | 97 |  |  | 0.92 | 0.46-1.83 | 5.7 | 0.303 |  |
|  | ICC | 1 | 43 |  |  | 1.48 | 0.48-4.59 | 0.0 | NA |  |
|  | IMCC | 2 | 93 |  |  | 1.4 | 0.33-5.86 | 76.5 | 0.039 |  |
|  | Subtotal | 3 | 136 |  |  | 1.38 | 0.58-3.28 | 54.2 | 0.113 |  |
|  | ICC | 1 | 43 |  | EFS | 1.38 | 0.44-4.29 | 0.0 | NA | 0.922 |
|  | IMCC | 2 | 93 |  |  | 2.42 | 1.30-4.50 | 62.1 | 0.104 |  |
|  | Indirect HRs | 1 | 39 |  |  | 4.28 | 1.69-10.83 | 0.0 | NA |  |
|  | Direct HRs | 2 | 97 |  |  | 1.47 | 0.75-2.88 | 0.0 | 0.895 |  |
|  | Subtotal | 3 | 136 |  |  | 2.12 | 1.23-3.66 | 40.4 | 0.187 |  |
| Necrosis sign | CT/ICC | 2 | 322 | Presence/Absence | OS | 1.68 | 0.62-4.51 | 85.1 | 0.01 | 0.124 |
|  | MR/IMCC | 2 | 365 |  |  | 3.84 | 1.77-8.31 | 70.7 | 0.065 |  |
|  | Subtotal | 4 | 687 |  |  | 2.57 | 1.21-5.46 | 89.9 | 0.001 |  |
|  | CT/ICC | 2 | 322 |  | EFS | 1.41 | 0.88-2.25 | 55.4 | 0.134 | 0.32 |
|  | MR/IMCC | 2 | 365 |  |  | 2.96 | 2.21-3.97 | 0.0 | 0.328 |  |
|  | Subtotal | 4 | 687 |  |  | 2.15 | 1.24-3.71 | 86.1 | 0.001 |  |
| Arterial phase enhancement pattern(3 Categories) | NA | 4 | 394 | Hypo/Hyper | OS | 5.90 | 3.40-10.00 | 8.0 | NA | NA |
|  |  |  |  | Rim/Hyper |  | 2.60 | 1.60-4.30 |  |  |  |
|  |  | 5 | 533 | Hypo/Hyper | EFS | 3.80 | 2.50-5.60 | 22.0 |  | NA |
|  |  |  |  | Rim/Hyper |  | 2.90 | 2.00-4.10 |  |  |  |

Table S3 Combined results of unadjusted HRs
